# Effects of Age, Season, Gender and Urban-Rural Status on Time-Activity: Canadian Human Activity Pattern Survey 2 (CHAPS 2)

**DOI:** 10.3390/ijerph110202108

**Published:** 2014-02-19

**Authors:** Carlyn J. Matz, David M. Stieb, Karelyn Davis, Marika Egyed, Andreas Rose, Benedito Chou, Orly Brion

**Affiliations:** 1 Air Health Effects Assessment Division, Health Canada, 269 Laurier Ave West, PL 4903C, Ottawa, ON K1A 0K9, Canada; E-Mail: marika.egyed@hc-sc.gc.ca; 2 Population Studies Division, Health Canada, 445-757 West Hastings Street—Federal Tower, Vancouver, BC V6C 1A1, Canada; E-Mail: dave.stieb@hc-sc.gc.ca; 3 Population Studies Division, Health Canada, 50 Colombine Driveway, Tunney’s Pasture, PL 0801A, Ottawa, ON K1A 0K9, Canada; E-Mails: karelyn.davis@hc-sc.gc.ca (K.D.); orly.brion@hc-sc.gc.ca (O.B.); 4 R.A. Malatest & Associates, Ltd., 858 Pandora Ave, Victoria, BC V8W 1P4, Canada; E-Mails: a.rose@malatest.com (A.R.); b.chou@malatest.com (B.C.)

**Keywords:** time-activity patterns, human exposure, population survey, exposure assessment, urban-rural

## Abstract

Estimation of population exposure is a main component of human health risk assessment for environmental contaminants. Population-level exposure assessments require time-activity pattern distributions in relation to microenvironments where people spend their time. Societal trends may have influenced time-activity patterns since previous Canadian data were collected 15 years ago. The Canadian Human Activity Pattern Survey 2 (CHAPS 2) was a national survey conducted in 2010–2011 to collect time-activity information from Canadians of all ages. Five urban and two rural locations were sampled using telephone surveys. Infants and children, key groups in risk assessment activities, were over-sampled. Survey participants (n = 5,011) provided time-activity information in 24-hour recall diaries and responded to supplemental questionnaires concerning potential exposures to specific pollutants, dwelling characteristics, and socio-economic factors. Results indicated that a majority of the time was spent indoors (88.9%), most of which was indoors at home, with limited time spent outdoors (5.8%) or in a vehicle (5.3%). Season, age, gender and rurality were significant predictors of time activity patterns. Compared to earlier data, adults reported spending more time indoors at home and adolescents reported spending less time outdoors, which could be indicative of broader societal trends. These findings have potentially important implications for assessment of exposure and risk. The CHAPS 2 data also provide much larger sample sizes to allow for improved precision and are more representative of infants, children and rural residents.

## 1. Introduction

Time-activity data are a key component of exposure assessment models used to estimate exposure to environmental contaminants. Microenvironment pollutant concentrations are combined with time-location information and activity-specific breathing rates to generate time-weighted exposure estimates. Human activity data have been incorporated into stochastic exposure models including the Hazardous Air Pollutant Exposure Model (HAPEM) [1], Air Pollutants Exposure Model (APEX) [2], and EXPOLIS [3]. Population exposure models that require a large quantity of input data, including time-activity data, may increase accuracy of exposure estimates and distributions compared to exposure estimates based solely on ambient air quality monitoring data [4]. Results of population exposure assessments may subsequently be used as a part of a risk assessment, cost-benefit analysis, or to support science-based policies or strategies to reduce exposures. 

Large-scale collections of time-activity pattern data have been conducted previously in North America, including the original Canadian Human Activity Pattern Survey (CHAPS) [5] and the National Human Activity Pattern Survey (NHAPS) [6] conducted in the US. Both these surveys were conducted in the early-mid 1990’s and collected 24-hour recall diaries using computer-assisted telephone interview (CATI) technology. Both surveys found that people spent the majority of daily time indoors, with some age-based differences in time-activity patterns. Comparison of the two surveys determined that with the exception of seasonal differences, location and time-activity patterns were very similar for Canada and the United States [7].

The main purpose of the present study, termed CHAPS 2, was to collect updated time-activity data, representative of the Canadian population, as they are key data to support population exposure and risk assessment activities. It has been over 15 years since CHAPS 1 and societal trends, including increased access to and use of personal computers [8], increased commute times [9], and increased teleworking or working from home [10,11] during this period may have affected human activity patterns. Also, efforts were taken to target sampling of infants and young children as these age groups were not well captured in CHAPS 1 and are key groups in risk assessment activities, as they are more vulnerable to environmental pollutants [12]. Additional objectives of CHAPS 2 were to assess, using up to date data, the effects of age, season, gender and urban-rural status, which were identified a priori as potentially important determinants of time-activity patterns. This paper describes the CHAPS 2 data collection methodology, sample characteristics, and main results.

## 2. Experimental Section

### 2.1. Selection of Subjects

The target population for CHAPS 2 was residents with a telephone living in the urban core of the Census Metropolitan areas of: Vancouver, British Columbia; Edmonton, Alberta; Toronto, Ontario; Montreal, Quebec; and, Halifax, Nova Scotia, as well as those living in the rural portions of the Census Divisions of: Haldimand-Norfolk, Ontario; and, Annapolis Valley-Kings County, Nova Scotia. The urban centres were chosen as they cover varied geographic and climatic regions, include three cities surveyed in CHAPS 1, and with a combined population of over 11,000,000 represent over 35% of the entire Canadian population. To increase the representativeness of the survey, rural regions were included in the sampled areas as almost 19% of the Canadian population resides in rural areas [13]. Both rural areas were located in fruit growing regions and had a combined population of over 100,000. Sampling also included francophone residents, as over 20% of the Canadian population identifies French as a first language [14], and CHAPS 1 was conducted in English only. The sample frame was split into two sampling sub-frames (infant and non-infant), as a means to best meet the objective of targeting infants (*i.e.*, respondents under 1 year of age). The non-infant survey entailed random selection of a household member older than 1 year of age, while the infant survey included only individuals less than 1 year of age.

For each location, the associated listed residential telephone numbers were identified from ASDE Survey Sampler Inc., which provided sampling frames based on up-to-date phone book listing information. These telephone numbers were also supplemented using random digit dialling (RDD) techniques. For RDD samples, a randomized sample of houses was developed using the “random B” methodology (a variant of the Mitofsky-Waksberg sampling). This process includes known phone numbers and randomly generates numbers that have a high probability of being valid for a residential household. This method generated samples that may have included cell phone numbers, to potentially capture cell phone-only households. To confirm that the geographic location of a respondent was within the defined area, respondents were asked to provide their postal code, which was used for verification of survey area. Screening questions were employed to confirm that rural respondents resided in the rural portion of the Census Division and to eliminate those in the urban areas. Distinct telephone number samples were drawn for the non-infant and infant surveys and for summer and winter sampling. Each sample was randomly divided into Survey A and Survey B strata (described below). Survey administration was divided into a summer sampling session (July to September 2010) and a winter sampling session (January to March 2011). A minimum of 20 call attempts was made to each number before it was considered non-responsive. A prize draw was used as an incentive for survey participation. Two prizes of $300 (one for each of the infant and non-infant surveys) were awarded per sampling season. 

For the non-infant survey, in households consisting of adults only (*i.e.*, 18 years and older), an adult was selected at random using the “next birthday” method. In this method, the interviewer inquires who will have the next birthday and selects that person. In order to oversample children, for households consisting of adults and children (*i.e.*, 1 to 17 years of age), a child was selected 70% of the time from all the children in the home and the remaining 30% of the time an adult was selected from all the adults in the home, also using the “next birthday” method. For any respondent under 10 years of age, the adult in the household most knowledgeable about the child’s or infant’s activities was requested to complete a proxy interview. For youth respondents aged 10-17 years, an adult in the household answered demographic and household questions while the youth completed the recall diary and post-diary activity questions about his/her own activities, unless the adult preferred to answer these questions on the youth’s behalf through a proxy interview. All interviewing of participants aged 17 years and younger was conducted with parental consent.

### 2.2. Survey Instrument

To maintain consistency and allow for comparison between cycles, the CHAPS 2 survey instrument was based on the CHAPS 1 surveys with some modifications. The survey instrument was comprised of three main components: questions regarding respondent characteristics and household composition; the 24-hour recall diary; and, a supplemental questionnaire regarding activities related to potential exposures to specific pollutants, dwelling characteristics, and socio-economic factors. The survey instrument is provided in the Supplemental Material.

For the 24-h recall diary section, the respondents were asked to describe what they had done yesterday, from midnight of the previous day to midnight last night. The CATI diary tool was designed to capture the amount of time, location, and activity for each sequential activity in the 24-h period. The location and activity information were matched to codes that had been used in CHAPS 1. Infant-specific activity codes were developed to facilitate the infant survey. Additionally, if a respondent indicated that an activity had taken place in a vehicle or constituted active transportation (e.g., walking, biking), supplementary questions regarding time spent in/near moderate to heavy traffic were asked.

Supplemental questions were divided between Survey versions A and B to reduce the respondent’s burden. The two versions had common core questions and unique groups of questions on specific topics. A similar approach to reduce the length of the interview was utilized in NHAPS and CHAPS 1.

### 2.3. Data Analysis

Survey weights were calculated to account for oversampling of certain age groups, post-stratification, to allow for combined analysis of the infant and non-infant survey, and to allow for generalization of survey results to the entire target population. Given the complexity of the survey design, weighting consisted of several steps: initial calculation of a basic weight; adjustments for non-response; adjustments for multiple telephone lines within a household; an adjustment for oversampling of individuals under the age of 18; removal of out of scope records; and, to ensure the population estimates were consistent with known region-age-sex totals from the Canadian Census population counts.

For statistical analysis, the survey weights were applied and procedures for survey data were utilized for generation of descriptive statistics, regression analysis, and ANOVA models to account for group differences. Data analysis was performed using SAS Enterprise Guide 4.2 (SAS Institute Inc., Cary, NC, USA) using SURVEYMEANS, SURVEYREG, and SURVEYFREQ procedures. Unless otherwise indicated, a significance level of α = 0.05 was implemented for hypothesis testing.

As this is the first paper presenting the survey results and to facilitate analysis of time-activity patterns, the location codes employed in CHAPS 2 were classified into four major groupings: indoors at home, other indoor locations, outdoors, and in vehicle. Grouping of location codes is provided in the Supplemental Material. For population exposure modeling, time-activity diary data are often collapsed into these or comparable groupings [15,16]. Overall time-activity patterns were determined by considering the weighted responses of all respondents. Seasonal, age-related, gender, and urban-rural differences in time-activity pattern were evaluated based on a priori hypotheses that these would be important determinants of time activity patterns. This study was approved by Health Canada’s Research Ethics Board.

## 3. Results

### 3.1. Survey Response

Survey response and refusal rates were determined using the methods outlined by Statistics Canada for random-digit dialing surveys [17,18]. Compared to other response rate calculation methods, this approach estimates the number of eligible and ineligible units from the unresolved telephone numbers at the end of the collection phase. Call distribution and survey response are summarized in Table 1. Rates are reported separately for the non-infant and infant surveys, due to differences in sampling design. The infant survey response and refusal rate estimates should be considered very conservative and interpreted with caution. Many of the refusals likely corresponded to households that would have been considered out-of-scope, as many households declined to participate in the survey prior to determination if the household had an infant. Given the low prevalence of infants in the general population, the majority of these unverified homes likely would not include an infant.

Efforts were made to sample each day of the week with similar frequency. The least number of surveys was collected for Saturday (recall survey completed on a Sunday) and the most surveys were collected for Tuesday (recall survey completed on a Wednesday). The weekday/weekend split was 72.4%/27.6% of weighted totals, which was close to the targeted split of 71.4%/28.6%.

**Table 1:** CHAPS 2 survey response by call distribution.

Call Distribution	Infant Survey		Non-Infant Survey	
	Number	%	Number	%
Telephone numbers drawn ^1^	53,634	-	54,642	-
Known non-households ^2^	11,034	-	13,817	-
Known not in-scope households ^3^	20,271	-	0	-
Known in-scope households ^4^	3,736	-	38,700	-
Unresolved numbers ^5^	18,593	-	2,125	-
Total in-scope households ^6^	5,573	-	40,268	-
Completed surveys ^7^	161	2.9 ^11^	4,850	12.0 ^11^
Refusals ^8^	3,409	91.2 ^12^	19,628	50.7 ^12^
No contacts ^9^	48	1.3 ^12^	11,728	30.3 ^12^
Other ^10^	688	18.4 ^12^	2,307	6.0 ^12^

Notes: ^**1**^ Telephone numbers drawn: total number of phone numbers drawn for the survey; **^2^** Known non-households: includes numbers that did not correspond to a household including not in service, business, and fax numbers; **^3^** Known not in-scope households: households contacted that did not meet eligibility requirements for the survey (*i.e.*, did not include an infant for the infant survey); **^4^** Known in-scope households: all households that met eligibility requirements for the survey; **^5^** Unresolved numbers: could not be determined if the number corresponded to a household that met eligibility requirements for the survey; **^6^** Total in-scope households: number of in-scope households based on known in-scope households and estimates from unresolved numbers; **^7^** Completed surveys: all households in which the selected participant completed the interview through the recall diary; **^8^** Refusals: contacted households which refused to participate in the survey or terminated the survey before completion of the recall diary; **^9^** No contacts: households in which an eligible participant was identified but never completed the survey (e.g., appointment was made but survey not completed), and for the non-infant survey included households in which only an answering machine was reached; **^10^** Other: households in which language and/or communication issues precluded survey completion; **^11^** % calculated based on total in-scope households; **^12^** % calculated based on known in-scope households.

### 3.2. Survey Sample Population

In total n = 5,011 surveys were completed. Sampling was fairly evenly distributed between the locations, with the most surveys collected from Edmonton and Annapolis Valley-Kings County and the least from Halifax. The distribution of respondents by age group and season, and a comparison to the target population are provided in Table 2. Efforts to oversample infants and children in CHAPS 2 were successful, as these groups were present at a greater proportion in the survey sample than in the target population. Seniors were also over-represented in the sample, while adolescents and adults were somewhat under-represented. Overall, more females (58.1%) than males (41.9%) completed the survey, though post-stratification weighting adjusted for the gender imbalance. Based on 2006 Canadian census data [19], CHAPS 2 respondents aged 25–64 were more likely than the general population in this age group to have a university degree at Bachelor level or higher (50.8% of the weighted CHAPS 2 respondents had a university degree or higher compared to 23% in the 2006 census). 

**Table 2:** Distribution of CHAPS 2 respondents by age and season.

Age Group	Number of Respondents			% of Sample	% of Target Population ^1^
	Summer	Winter	Total		
Infants (<1 year)	81	80	161	3.2	1.1
Young children (1–4 years)	132	137	269	5.4	4.5
Children (5–11 years)	217	211	428	8.5	7.6
Adolescents (12–19 years)	177	153	330	6.6	9.1
Adults (20–59 years)	1,081	1,114	2,195	43.8	59.5
Seniors (60+ years)	818	810	1,628	32.5	18.1
Total	2,506	2,505	5,011	100	100

Note: ^**1**^ Based on Canadian Census 2011 [20].

### 3.3. Time-activity Patterns

The 24-h recall diary information was used to estimate time-activity patterns for the target Canadian population, combining both non-infant and infant survey data. An overall time-activity pattern, representing both seasons and all age groups, is presented in Table 3. On average, a large portion of the day was spent in indoor locations. Combining time spent indoors at home and in other indoor locations, over 21 h (or 88.9% of a day) was spent indoors. Of the time spent at home, sleeping/napping accounted for slightly more than half this time (35.9% of a day), leaving just over 8 waking hours spent at home. Almost half of the daily time spent at other indoor locations was attributable to time in work/school (8.7% of daily time), while much smaller portions of time were spent in mall/store (2.0% of daily time) and restaurant/bar (1.2% of daily time) locations. Relatively smaller portions of the day were spent outdoors and in vehicle. Of the time spent in vehicle, almost 1 h was in a personal vehicle (4.1% of daily time) while very little time was spent in a bus (0.6% of daily time).

**Figure 1:**
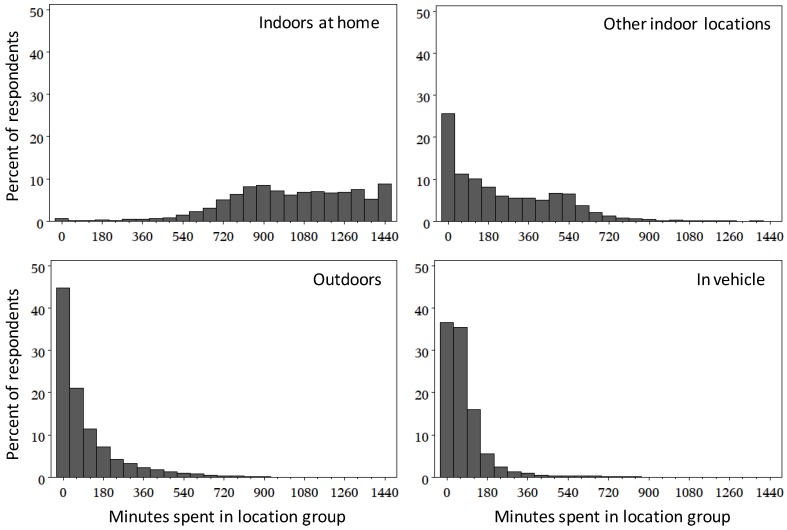
Distributions of time spent indoors at home, other indoor locations, outdoors, and in vehicle for all CHAPS 2 respondents.

From the data provided in Table 3, the mean and median of time spent indoors at home were quite similar, while for the other location groups, the mean was greater than the median indicating a right-skew to the distribution of time spent in these locations (Figure 1). Nearly all participants indicated spending time indoors at home during the diary day, with a majority of the respondents spending at least 600 min at home. In comparison, 21.8% of respondents did not indicate spending any time in other indoor locations, 30.9% did not spend any time outdoors, and 24.1% of participants did not spend time in vehicle. Compared to the indoor location groups, much less variability was noted in the distributions for outdoors and in vehicle, with the majority of respondents indicated spending 60 min or less in either of these locations. 

**Table 3:** Daily time spent in different locations for the target population.

Location	Mean			Standard Error			Median			95th Percentile	
	% of day	h:min		% of day	min		% of day	h:min		% of day	h:min
Indoors at home	69.9	16:46		0.6	8.7		69.7	16:44		99.9	23:58
Other indoor locations	19.0	4:33		0.6	8.3		14.6	3:30		47.2	11:19
Outdoors	5.8	1:23		0.3	3.7		2.1	0:30		26.0	6:14
In vehicle	5.3	1:16		0.3	4.2		3.5	0:50		16.8	4:01

### 3.4. Influence of Season on Time-activity Patterns

By sampling in summer and winter months only, CHAPS 2 was designed to capture extremes of seasonal effects on daily time-activity patterns (Table 4). Season was a significant predictor of time spent indoors at home (*p* < 0.0001) and outdoors (*p* < 0.0001). Not unexpectedly based on weather conditions, the amount of time spent outdoors in the summer was more than double that in the winter, an average increase of 1 h 19 min spent outdoors. Correspondingly, an increase in time spent indoors at home during the winter was reported, representing an average increase of 1 h 11 min. In comparison, time spent in other indoor locations was not significantly different between the seasons. Although overall time in this location group did not vary with the seasons, the contribution of time spent in work and school to the other indoor locations group increased from 38.6% in the summer to 52.7% in the winter (*p* < 0.0001), reflecting differences in regular daily activities between the seasons (e.g., school and work activities in the winter compared to summer holidays). The seasonal effect on time spent in vehicle was not significant (*p* = 0.0517).

**Table 4:** Daily time spent in major locations for the population by season.

Season	Location	Mean		Standard Error			Median		95th Percentile	
		% of day	h:min	% of day	min	% of day		h:min	% of day	h:min
Summer	Indoors at home	67.5	16:11	0.9	13.5	68.0		16:19	97.7	23:27
	Other indoor locations	18.0	4:21	0.9	13.2	12.5		3:00	49.0	11:45
	Outdoors	8.6	2:03	0.5	6.9	4.1		0:59	33.3	8:00
	In vehicle	5.8	1:24	0.5	7.6	3.4		0:50	21.7	5:12
Winter	Indoors at home	72.4	17:22	0.7	10.5	72.4		17:21	99.9	23:58
	Other indoor locations	19.9	4:46	0.7	10.1	17.3		4:09	46.7	11:13
	Outdoors	3.0	0:44	0.2	3.1	0.8		0:11	12.5	2:59
	In vehicle	4.7	1:08	0.2	3.4	3.5		0:50	13.5	3:15

### 3.5. Influence of Age on Time-activity Patterns

Time-activity distributions by age group are presented in Table 5. Age was a significant predictor of time spent indoors at home (*p* < 0.0001). Compared to all other age groups, infants spent the most time indoors at home, averaging over 21 h a day (*p* < 0.0001). With increasing age, the general trend was a decrease in time spent indoors at home with adults spending the least amount of time indoors at home (*p* < 0.0001 compared to infants-children). Comparing adults and seniors, seniors spent significantly more time indoors at home, an increase of 2 h 36 min daily (*p* < 0.0001). For all age groups a large portion of the time indoors at home was spent sleeping/napping, ranging from 45% (seniors) to 64% (young children) of daily time spent indoors at home.

Age was also a significant predictor of time spent in the other main location groups: other indoor locations (*p* < 0.0001); outdoors (*p* = 0.0174); and, in vehicle (*p* < 0.0001). Compared to the other age groups, infants spent the least amount of time in other indoor locations (*p* < 0.0001). For young children, children, adolescents, and adults, about half of the time spent in other indoor locations was in work and/or school. When considering time spent outdoors, at about 2 h per day on average, young children and children spent the most time in this environment; however, differences between the age groups were not statistically significant. Of the age groups, adults spent the most time in a vehicle (*p* < 0.0001 compared to infants-adolescents; *p* = 0.0003 compared to seniors) and were the only group to on average spend more time in a vehicle than outdoors. For all age groups, time spent in a personal vehicle was the main contributor to time spent in a vehicle, ranging from 65.8% for adolescents to 93.6% for young children. 

**Table 5:** Daily time spent in major locations by age group.

Age Group	Location	Mean			Standard Error			Median			95th Percentile	
		% of day	h:min		% of day	min		% of day	h:min		% of day	h:min
Infants (<1 year)	Indoors at home	89.2	21:23		1.5	21.1		93.3	22:22		99.9	23:58
	Other indoor locations ^1^	4.8	1:10		0.9	13.4		0.0	0:0		23.6	5:39
	Outdoors ^1^	4.0	0:57		1.1	16.0		0.0	0:0		21.2	5:06
	In vehicle ^1^	2.0	0:29		0.3	5.0		0.0	0:0		8.7	2:05
Young Children (1–4 years)	Indoors at home	74.0	17:44		1.6	22.9		71.6	17:10		98.0	23:30
	Other indoor locations	15.3	3:40		1.5	21.0		9.6	2:19		39.4	9:23
	Outdoors ^1^	7.6	1:49		1.3	18.9		4.0	0:58		31.5	7:33
	In vehicle ^1^	3.2	0:46		0.6	8.0		1.6	0:22		8.3	1:59
Children (5–11 years)	Indoors at home	71.3	17:07		1.2	17.8		70.1	16:48		95.2	22:50
	Other indoor locations	17.8	4:16		1.1	16.3		18.1	4:21		38.9	9:20
	Outdoors	7.5	1:48		0.6	8.1		4.9	1:11		24.7	5:55
	In vehicle	3.4	0:49		0.4	5.4		2.1	0:30		11.8	2:50
Adolescents (12–19 years)	Indoors at home	69.5	16:40		2.0	28.9		69.2	16:36		98.3	23:35
	Other indoor locations	20.8	4:59		1.8	26.2		22.2	5:19		44.8	10:44
	Outdoors	6.2	1:29		1.0	14.8		1.4	0:20		29.9	7:10
	In vehicle	3.5	0:50		0.3	4.9		2.4	0:34		10.4	2:29
Adults (20–59 years)	Indoors at home	66.8	16:02		0.9	12.5		64.5	15:28		99.1	23:45
	Other indoor locations	21.4	5:08		0.9	12.4		16.9	4:03		50.5	12:06
	Outdoors	5.5	1:19		0.4	5.4		2.0	0:29		25.9	6:12
	In vehicle	6.3	1:30		0.5	6.7		4.3	1:02		22.7	5:27
Seniors (60+ years)	Indoors at home	77.7	18:38		0.9	12.3		81.9	19:38		100.0	24:00
	Other indoor locations	12.5	2:59		0.7	10.2		8.0	1:55		40.6	9:44
	Outdoors	5.5	1:19		0.3	4.9		2.0	0:29		24.6	5:54
	In vehicle	4.4	1:03		0.2	3.5		2.4	0:35		13.9	3:20

Note: ^**1**^ High sampling variability associated with the estimates, interpret with caution.

### 3.6. Influence of Gender on Time-activity Patterns

Time-activity distributions by gender are presented in Table 6. Gender was a significant predictor of daily time spent indoors at home (*p* = 0.0118) and outdoors (*p* = 0.0164). More daily time was spent indoors at home by females, an average of about 44 min more than males. In comparison, males spent almost 20 min more time outside compared to females. 

**Table 6:** Daily time spent in major locations for the population by gender.

Gender	Location	Mean			Standard Error			Median			95th Percentile	
		% of day	h:min		% of day	min		% of day	h:min		% of day	h:min
Male	Indoors at home	68.4	16:24		0.9	13.2		67.5	16:12		99.5	23:52
	Other indoor locations	19.8	4:44		0.9	12.8		15.3	3:41		47.2	11:19
	Outdoors	6.4	1:33		0.4	5.8		2.7	0:40		30.4	7:17
	In vehicle	5.4	1:18		0.5	7.6		3.7	0:20		16.7	4:00
Female	Indoors at home	71.4	17:08		0.8	11.2		72.4	17:22		99.9	23:58
	Other indoor locations	18.3	3:56		0.7	10.5		13.8	3:19		46.9	11:15
	Outdoors	5.2	1:14		0.3	4.8		1.4	0:20		21.8	5:14
	In vehicle	5.1	1:14		0.3	3.9		3.4	0:49		17.3	4:09

### 3.7. Influence of Urban-rural Status on Time-activity Patterns

Time-activity distributions for urban and rural populations are presented in Table 7. Living in a rural location was a significant predictor of daily time spent outdoors (*p* < 0.0001) and in other indoor locations (*p* = 0.0011). People living in rural areas spent about 1.7-fold more time outdoors compared to people living in urban areas, an increase of 58 additional minutes outside per day. This observed increase in time spent outdoors for the rural population was associated with a reduction in time spent in other indoor locations, especially work and school environments, compared to the urban population. On average, people living in rural areas spent 35 min less per day in work and/or school locations compared to those living in urban areas (*p* < 0.0001). No significant differences were noted in time-activity pattern between the urban locations.

**Table 7:** Daily time spent in major locations for the population by urban-rural status.

Status	Location	Mean		Standard Error		Median		95^th^ Percentile	
		% of day	h:min	% of day	min	% of day	h:min	% of day	h:min
Urban	Indoors at home	70.0	16:47	0.6	8.8	69.8	16:44	99.7	23:55
	Other indoor locations	19.0	4:34	0.6	8.3	14.6	3:30	47.2	11:19
	Outdoors	5.8	1:23	0.3	3.8	2.1	0:30	26.0	6:14
	In vehicle	5.3	1:16	0.3	4.2	3.5	0:50	16.8	4:01
Rural	Indoors at home	68.2	16:22	0.9	12.7	67.0	16:04	99.9	23:58
	Other indoor locations	16.0	3:50	0.7	10.4	9.7	2:20	47.7	11:26
	Outdoors	9.8	2:21	0.5	7.9	4.2	1:00	39.1	9:23
	In vehicle	6.0	1:26	0.4	5.4	3.8	0:54	17.4	4:10

## 4. Discussion

### 4.1. Time-activity Patterns

Time-activity patterns provide key information for population exposure modeling. Comparing exposure estimation approaches, a recent reported that exposure estimates were refined by incorporation of time-activity patterns, and that relying only on a central site monitor could result in exposure misclassification [21]. In this study, several factors were determined to have a significant effect on time-activity patterns, including season, age, gender, and urban-rural status. 

From analysis of the CHAPS 2 recall diary data several general observations are evident, which could have impacts on exposure assessments. A large majority of time was spent indoors at 88.9% overall (69.9% at home and 19.0% in other indoor locations) and substantial amounts of daily time were spent at home by infants and seniors. A similar skew was noted in NHAPS, with many respondents spending long periods of time indoors, mainly in a residence [6]. Given the proportion of time spent in these locations, potential exposures in indoor microenvironments would be expected to contribute substantially to an overall exposure estimate. Average daily time spent outdoors was 5.8%; this was greater for young children, children, and adolescents, and for the rural population. Similar age-related differences in time-activity also were noted in CHAPS 1 [5]. For these groups, potential exposure from outdoor sources of pollutants would be expected to have a greater contribution. Time spent in vehicle represents 5.3% of a day, and was greatest for adults. The large majority of time spent in vehicle was associated with travelling in a personal vehicle. Although the time spent in vehicle is small compared to time spent indoors, the higher concentration of traffic-related air pollutants in this microenvironment may contribute significantly to an overall exposure estimate. 

Seasonal differences in time-activity patterns were also noted in CHAPS 1 (*i.e.*, Canadian) and NHAPS (*i.e.*, American) [7]. In these previous studies, both Canadians and Americans spent at least twice as much time outdoors in the summer compared to the winter. Time spent in work/school locations during the winter, compared to the summer, was greatly increased in respondents less than 17 years of age. A more recent time-activity study conducted in California also reported a seasonal effect, with increased time spent at home by children and in offices for all respondents, and decreased time spent in parks during the cool season for all respondents [22].

While some time-activity patterns were similar to previous studies, other potentially important changes were also evident. Comparing adult respondents (>17 years in CHAPS 1 and ≥20 years in CHAPS 2), although the time spent indoors was similar, in the current study the average time spent indoors at home by adults and seniors was slightly greater, at 69.4% compared to 64.3% in CHAPS 1 or an increase of about 1 h 13 min per day, with less time spent in other indoor locations. The group of other indoor locations is quite diverse, encompassing all indoor locations that are not the respondent’s home. As such, the observed overall decrease in time spent in this location group most likely reflects many small changes in time-activity patterns. For example, a small decrease in time spent in mall/store microenvironments was observed for adults and seniors in CHAPS 2 (2.2% of daily time) compared to CHAPS 1, at 3.1% of time [7].

Other changes in time-activity patterns observed since CHAPS 1 included a small increase in time spent indoors at home for the youth respondents of CHAPS 2. For example, adolescent respondents in CHAPS 2 (12–19 years) averaged 69.5% of time indoors at home compared to 67.8% for youth respondents in CHAPS 1 (11–17 years), a difference of 24 min; correspondingly, adolescents in CHAPS 2 spent less time outdoors at 6.2% compared to 8.6% in CHAPS 1, a difference of almost 35 min per day. Overall, these results indicate that there are measurable differences in time-activity patterns for certain age groups which are consistent with broader societal trends such as increased time spent teleworking or working from home [10,11] and possibly increased screen-time by adolescents [23,24], potentially at the expense of time spent outdoors. A trend of decreasing time spent outdoors has been noted in time-activity patterns of Americans over the past few decades, indicating population behavior shift [25]. Although small, changes in time-activity pattern may influence exposures as daily time in different microenvironment changes, altering the relative contribution of different sources to overall exposure.

In this study, differences were noted in time-activity patterns for males and females. A California study of time-activity patterns noted some differences in time spent in different microenvironments between males and females [22]. For examples, males reported more time in public parks and health clubs, while females spent more time in school and at food stores. 

Rural respondents spent more time outdoors and less time indoors compared to their urban counterparts. These differences are reflective of the anticipated differences in occupational and leisure activities between agricultural rural environs and large urban centers. Prior to CHAPS 2, there was only a limited indication that people in rural areas spend more time outside compared to people living in urban centers [26]. A greater proportion of time spent outdoors by people living in rural areas, potentially coupled with increased physical activity levels associated with agricultural and related occupations, would result in increased exposure to/dose of outdoor air pollutants and other outdoor physical, chemical and biological agents. Indeed, preliminary analysis of CHAPS 2 questionnaire data indicates that rural respondents were more likely to report working with or near gasoline or diesel powered equipment, solvents, and pesticides. Geographic variability in exposure due to differences in time-activity data between study locations has been demonstrated previously [21].

Distributions of time spent in other indoor location, outdoors, and in vehicle were skewed to the right, with many respondents not spending time in at least one of these location groups. Considering that CHAPS 2 and the preceding NHAPS and CHAPS 1 employed RDD strategies to contact participants for telephone interviews, it is possible that people who spend more time at home would be home to answer the telephone. The SUPERB study conducted in California collected 24-hour recall diary information via web surveys, which could be completed at any time convenient for the respondent, and telephone surveys [27]. Time spent indoors at home was similar between web- and telephone-based surveys, and similar to the results of CHAPS 2, suggesting that telephone-based surveys may not necessarily over-estimate time spent indoors at home. This study also reported skewed distributions of time spent in different microenvironments, with nearly all participants reporting time at home and many diaries reporting no time spent in particular microenvironments [22].

### 4.2. Sample Representativeness

The representativeness of the survey sample determines the applicability or ability to generalize the results to the target population. Improved representativeness of the CHAPS 2 survey compared to CHAPS 1is gained from the sampling of urban and rural centers across the country, all age groups, Francophones and Anglophones, and during both summer and winter in all locations. Other factors impacting representativeness include response rates and how well the socio-demographic characteristics of the sample correspond to the population. We employed a modest incentive in an attempt to maximize the response rate. Nonetheless, response rates have been decreasing in telephone surveys for the past several decades, with steeper declines in the more recent past [28,29]. Declining response rates have been associated with increased use of caller ID and answering machines [30]. Decreasing response rates may introduce a bias if respondents have different characteristics than non-respondents for variable(s) of interest. Indeed, a greater proportion of CHAPS 2 respondents (aged 25–64) had a university or higher level of educational attainment compared to the Canadian population of the same age. A similar observation was made in CHAPS 1, despite a much higher response rate. However, low response rates do not necessarily result in non-response bias [31], and telephone surveys with proper weighting have been demonstrated to provide accurate information, despite low response rates [29]. To limit the impact of non-response bias and expand the applicability of the results to the target population, weighting included adjustments for non-response and to account for targeted sampling of infants and children. Given the difficulties with declining response rates and such design requirements, future studies could consider supplementing random digit dialing procedures with additional survey frame information to assist in subject identification and retention. 

## 5. Conclusions

Time-activity data are essential input parameters for estimating the range of personal exposures to environmental pollutants in a population or subset(s) of the population. Estimates of population exposure are improved with use of time-activity patterns to account for the variability in exposure between locations compared to estimates based on central site monitors alone. Season, age, gender, and urban-rural status were significant predictors of time activity patterns. Compared to CHAPS 1 data from 15 years ago, adults reported spending more time indoors at home and adolescents reported spending less time outdoors, which could be indicative of broader societal trends. Changes in time-activity pattern may influence exposures as daily time in different microenvironment changes, altering the relative contribution of different sources to overall exposure. These new results will inform exposure and risk assessment activities pertaining to environmental pollutants.

## Supplementary Files

Supplementary File 1 Supplementary Materials (PDF, 1100 KB)
