# Supplementary material for: Effects of Age, Season, Gender and Urban-Rural Status on Time-Activity: Canadian Human Activity Pattern Survey 2 (CHAPS 2)
Source: Int J Environ Res Public Health. 2014 Feb 19;11(2):2108–24. doi: 10.3390/ijerph110202108 (PMC3945588; doi:10.3390/ijerph110202108)
Supplement: Supplementary File 1 — Supplementary Materials (PDF, 1100 KB) [file ijerph-11-02108-s001.pdf]

Supplemental Material

Appendix A – CHAPS 2 Survey Instrument
Non-Infant Questionnaire pg. 2

Infant Questionnaire pg. 37

Appendix B – CHAPS 2 Activity and Location Codes pg. 63

Appendix C – Location Groupings pg. 69

Appendix A – CHAPS 2 Questionnaires

Non-Infant Survey

CHAPS 2 - NON-INFANT COMBINED FORM A & B QUESTIONNAIRE

ALL – Applicable to all non-infant survey type and both form A and B
ADULT – Applicable to all adult survey type and both form A and B
CHILD – Applicable to all child survey type and both form A and B
NONPROXY – Applicable to all adult and child direct and both form A and B
FORMA – Applicable to all survey type but only form A
FORMB – Applicable to all survey type but only form B

Code	Survey Type	Form
ALL	Non-Infant	A & B
ADULT	Adult Only	A & B
CHILD	Child Direct Only	A & B
NONPROXY	Adult + Child Direct Only	A & B
FORMA	Non-Infant	A
FORMB	Non-Infant	B

ALL

INTRODUCTION BLOCK START

INTRO1

For all locations other than Montreal:
Hello, my name is ____. I'm calling for Health Canada.

Bonjour, je m'appelle _____. J'appelle de la part de Santé Canada.

Would you prefer to continue in English or French?

For Montreal:
Bonjour, je m'appelle _____. J'appelle de la part de Santé Canada.

Hello, my name is ____. I'm calling for Health Canada.

Préférez-vous continuer en anglais ou en français?

Remainder to be conducted in language of choice.

** IF YOUNG CHILD ANSWERS, ASK TO SPEAK WITH AN ADULT

We are conducting a survey to assess Canadians' exposure to pollutants. Your responses will inform Health Canada on how to protect the health of Canadians.

If you complete the survey, you can enter a draw to win a cash prize of $300. The draws will take place on September 30, 2010 (*summer*) March 31, 2011 (*winter*).

[Surveyor: If asked about odds of winning, read] Your chance of winning is approximately 1 in 2400.]

Would you be able to do the survey with me?

	If no, read<br><br>While participation is voluntary, your cooperation is important to ensure the information collected is as accurate as possible. Your participation is also important because your response will represent not only your household, but similar households in your area.<br><br>Would you like to take part in this study?<br><br>If yes, continue:<br><br>Thank you. The study will update information collected 15 years ago. The survey asks you to recall your activities from the previous day and asks specific questions relating to your home. Knowing where and how Canadians spend their time will help Health Canada to estimate Canadians' exposure to pollutants. These estimates help set standards to protect the health of Canadians.<br><br>Participation is voluntary and you can skip over questions you do not want to answer. Your name and other identifying information will not be associated with your responses and any information you provided will be used for research purposes only and will be kept strictly confidential.<br><br>Before we begin, would you have time for me to ask you a few questions to determine which person in your household should complete the survey?<br>[Surveyor: if asked, read] The survey selects one respondent depending on who lives in your household and who next celebrate his or her birthday.<br>[Surveyor: If asked about survey length, read]<br>The survey can be completed in 20 minutes or less.<br><br>[If pressed, read] Depending on how you answer each question, it may take you more or less time to go through the survey.<br><br>[1] YES (BEGIN SURVEY) *[Qnadult]*<br>[2] Not now – ask for call-back time<br>[99]Refused (will terminate survey) *[Thanks and Terminate]*<br><br>**INTRODUCTION BLOCK END**
ALL	**SCREENING/STATS BLOCK START**<br><br>First, I have a few questions about the composition of your household.<br><br>QNADULT<br>Including yourself, how many adults, age 18 and older, live in your household?<br>[1] RECORD ACTUAL NUMBER *[nkid1]*<br>[11] MORE THAN 10 *[nkid1]*<br>[99] REF *[nkid1]*<br><br>NKID1<br>How many children under 18 live in your household?<br>[1] NONE *[INT2]*<br>[2] ONE *[Qkid]*<br>[3] RECORD NUMBER *[Qkid]*<br>[11] MORE THAN 10 *[Qkid]*

	[99] REF *[Qkid]*<br><br>QKID<br>What is the age of the youngest child living in your household?<br>[1] LESS THAN 1 *[Qninfant]*<br>[2] RECORD AGE 1 THRU 17 *[RANDOM1]*<br>[99] DK/REF *[RANDOM1]*<br>[loc 3/62]<br><br><SKIP Qkid2 if NIKD1 < 2><br><br>QKID2<br>Aside from your infant(s), what is the age of the youngest child living in your household?<br>[1] only infants in household *[Qninfant]*<br>[2] RECORD AGE 1 THRU 17 *[RANDOM1]*<br>[99] DK/REF *[RANDOM1]*<br><br>QNINFANT<br>Just to confirm, how many infants under 1 year live in your household?<br>[1] RECORD NUMBER *[RANDOM1]*<br>[99] REF *[RANDOM1]*
ALL	**IF total number of kid = total number of infant, go to INT2 (ADULT SURVEY)**<br>**ELSE**<br>**RANDOM 1 determined the 70/30 split for Adult or Child Survey**<br>**IF RANDOM1 > 71, go to INT2 (ADULT SURVEY ROUTE)**<br>**IF RANDOM1 <= 71, go to KNAM (CHILD SURVEY ROUTE)**
ADULT	**ADULT SURVEY ROUTE**<br><br>INT2 (ADULT SURVEY)<br>For this study I need to speak with the adult living in your household who is 18 or older, and will have the NEXT birthday. Who would that be?<br><br>(Surveyor: If R asked about the birthday selection method, say this is to ensure random selection of survey respondents).<br><br>SURVEYOR: IF INFORMANT DOES NOT KNOW ALL THE BIRTHDAYS ASK: Of the ones you do know, who will have the NEXT birthday?<br><br>[1] INFORMANT HAS NEXT BIRTHDAY (BEGIN SURVEY) *[Rsex]*<br>[2] SOMEONE ELSE HAS NEXT BIRTHDAY (ask for that person and repeat introduction) *[Repeat intro]*<br><br>RSEX<br>RECORD "ADULT" RESPONDENT'S SEX<br>[1] MALE *[Rage]*<br>[2] FEMALE *[Rage]*<br><br>RAGE<br>In what year were you born?

	[xxxx] RECORD ACTUAL YEAR *[PHONE1]*<br>[88] DK/REF *[PHONE1]*
CHILD	**CHILD SURVEY ROUTE**<br><br>KNAM (CHILD SURVEY)<br><br>**If only 1 child between the age of 1 to 17**<br>For this study, we need to know about the activities of the child in your household. I just need his or her first name.<br><br>**Else**<br>For this study, we need to know about the activities of the child in the household older than 1 year and under the age of 18 who will have the NEXT birthday. Who would that be? I just need his or her first name.<br>[1] RECORD CHILD'S FIRST NAME<br>[99] Refused<br>** DO NOT END WITH ///<br>|- - - - - - - - - - - -| *[Rage]*<br><br>KAGE<br><DISPLAY IF NKID1 = 1><br>You said that you had one child who was [age] years old. Can I confirm that child is [fill name]?<br>OR<br><DISPLAY IF NKID1 > 1><br>How old is [fill name]?<br>[1] LESS THAN ONE [Child should be older than 1 - hit "previous" and correct the information)] *[ksex]*<br>[2] RECORD AGE (1-17) *[ksex]*<br>[3] Yes, Child is [age] years old *[ksex]*<br>[88] REF/DK *[ksex]*<br><br>KSEX<br>** IF NOT SURE ASK: Is [fill name] a boy or girl?<br>[1] BOY *[wkid]*<br>[2] GIRL *[wkid]*<br>[99] REF *[wkid]*<br><br>**If child who has next birth date is:**<br>**1. between age 1 and 10, go to CHILD PROXY SURVEY**<br>**2. age 10 or older but younger than age 14 go to PROXY**<br>**3. age 14 and older, go to CHILD DIRECT SURVEY**<br><br>PROXY<br><DISPLAY if child is age 10 to 13><br>[*fill-name or your child*] is old enough to complete a portion of the survey questions [*fill your*]. You may listen on another line or stand beside [*him or her*], or, if you prefer, you can answer the survey on [*fill your*] behalf.<br><br><DISPLAY if child is age 10 to 17>

	Will you provide permission for [*fill-name or your child*] to complete portion of the interview on [*fill your*] own?<br><br><DISPLAY if child is age 10 to 13><br>Or would you prefer to complete this portion of the survey on [*fill your*] behalf?<br><br>[1] Consent for child to complete *[wkid]*<br>[2] Prefer to complete on child's behalf *[wkid] <display if child is age 10 to 13>*<br>[99] Refusal to involve child (will terminate survey *[Explain importance of survey;* *Convince respondents to change their mind; if failed, terminate]*<br><br>*Surveyor: If respondent will not provide consent, use the following text:*<br>*We cannot proceed with the survey if you do not provide consent or permission to interview your child. Your child has been selected to participate because it provides important information for Health Canada to accurately estimate the amount of environmental exposure to Canadians. The information will in turn help reduce the exposure of children to pollution.*<br><br>*If you provide permission for your child to complete the survey, we will be asking questions about how and where your child spent his or her time yesterday and about their proximity to pollutants.*<br><br>*Surveyor: If respondent is concerned about privacy and/or confidentiality, read:*<br>*Any information collected will be kept strictly confidential and answers will be used for the purpose of estimating Canadian exposure to pollution only. Your child will not be identified after the survey is completed. Information about your child's demographic are collected for statistical purposes only.*<br><br>WKID<br>This survey looks at children's exposure to different kind of environments. We will be asking questions about the child's activities yesterday.<br><br>I will need to speak with an adult, who lives in the household who would know how [fill name-child] spent [fill your] time yesterday. Who would that be?<br>[1] INFORMANT *[CDI]*<br>[2] SOMEONE ELSE *[HOME]*<br>[99] Refused (will terminate survey) *[Thanks and Terminate]*<br><br>**IF CHILD PROXY SURVEY and child is under 6 year old, SKIP HOME**
ALL	**HOME**<br>During this interview I will need to have both [fill know-adult] and [fill name-child] available to complete the survey. Are both [fill know] and [fill name] home now? [loc 0/31]<br><br>SURVEYOR: Introduction for new person, if necessary:<br>We are conducting a study to help reduce the exposure of both adults and children to pollution. Your answers are for statistical purposes only, and will be kept strictly confidential.<br><br>This interview is completely voluntary. If we come to any question you don't want to answer, just tell me and we'll skip it.

May I start the survey with you?

[1]	YES (Both)	*[PHONE1]*
[3]	YES (Child Only)	[Explain Informant need to be available *Call Back]*
[4]	YES (Adult Only)	*[PHONE1 if CHILD PROXY; Call Back if CHILD DIRECT]*
[2]	Both NO	[Explain Informant need to be available *Call back*]`
[99]	Refused(will terminate survey)	*[Explain importance of survey; Convince R to change their mind; if failed, terminate]*

**if home = 1, say: I now need to speak with [fill know-adult], please. [go to PHONE1]

CDI
During this interview I will need to have [fill name-child] available to complete part of the interview. Is [fill name] home?

SURVEYOR: Introduction for [your child], if necessary for CHILD DIRECT SURVEY:

We are conducting a study to help reduce the exposure of both adults and children to pollution. Your answers are for statistical purposes only, and will be kept strictly confidential.

This interview is completely voluntary. If we come to any question you don't want to answer, just tell me and we'll skip it.

[1]	NO	*[PHONE1 if CHILD PROXY; Explain child need to be available, Call Back If CHILD DIRECT]*
[2]	YES	*[PHONE1]*
[99]	Refused (will terminate survey)	*[Explain importance of survey; Convince respondents to change their mind; if failed, terminate]*

KREL
What is your relationship to [fill name]?

[1]	MOTHER	*[PHONE1]*
[2]	FATHER	*[PHONE1]*
[3]	OTHER - SPECIFY: [specify]	*[PHONE1]*
[99]	REFUSED	*[PHONE1]*

PHONE1
All together, how many different landline phone NUMBERS does your household have for non-business use?
SURVEYOR: If respondent does not know what a landline is, read: A land line is a regular telephone line, typically served over a pair of copper wires from a phone company into your home in a fixed location. They are named as such because they are connected to a physical line rather than a cell tower or satellite. It is not a cell phone or mobile phone.

[1]	No landlines
[2]	RECORD ACTUAL NUMBER
[6]	6 OR MORE

	[99] REF<br><br>PHONE2<br>All together, how many different cell phone NUMBERS does your household have for non-business use?<br>[1] No cell phones<br>[2] RECORD ACTUAL NUMBER<br>[6] 6 OR MORE<br>[99] REF<br><br>**[ASK SCREENING BLOCK ONLY IF REGION=6 {Annapolis Valley} OR REGION=7 {Haldimand Norfolk}]**<br><br>SCREENING1<br>What county is your home located in? [If necessary, read county list]<br>[ALTERNATE WORDING IF ONLY ONE COUNTY: Is your home located within...? READ COUNTY NAME]<br><br>[DISPLAY OPTIONS IF REGION=6]<br>[11] Annapolis County *[Go to SCREENING 2]*<br>[12] Kings County *[Go to SCREENING 2]*<br>[DISPLAY OPTIONS IF REGION=7]<br>[31] Haldimand County *[Go to SCREENING 2]*<br>[32] Norfolk County *[Go to SCREENING 2]*<br>[ALWAYS DISPLAY]<br>[77] Another county (please specify): ______________ *[TERMINATE SURVEY]*<br>[88] Don't Know *[Go to SCREENING 2]*<br>[99] Decline to Respond *[TERMINATE SURVEY]*<br><br>[If selected valid County in SCREENING1, list 'exclusion towns' within the sample region, as well as a few towns just outside the boundaries of the County that are excluded as well]<br><br>SCREENING2<br>Is your home located in a rural area? or within one of the following towns? [Read list]<br>[DISPLAY OPTIONS IF REGION=6]<br>[11] Bridgetown *[Go to SCREENING 3]*<br>[12] Middleton *[Go to SCREENING 3]*<br>[13] Digby *[Go to SCREENING 3]*<br>[14] Kingston *[Go to SCREENING 3]*<br>[15] Greenwood *[Go to SCREENING 3]*<br>[16] Berwick *[Go to SCREENING 3]*<br>[17] Coldbrook *[Go to SCREENING 3]*<br>[18] Kentville *[Go to SCREENING 3]*<br>[19] Hantsport *[Go to SCREENING 3]*<br>[DISPLAY OPTIONS IF REGION=7]<br>[20] Windsor *[Go to SCREENING 3]*<br>[21] Brantford *[Go to SCREENING 3]*<br>[22] Burtford *[Go to SCREENING 3]*<br>[23] Caledonia *[Go to SCREENING 3]*<br>[24] Cayuga *[Go to SCREENING 3]*<br>[25] Delhi *[Go to SCREENING 3]*<br>[26] Dunnville *[Go to SCREENING 3]*<br>[27] Hagersville *[Go to SCREENING 3]*

	[28] Jarvis *[Go to SCREENING 3]*<br>[29] Norwich *[Go to SCREENING 3]*<br>[30] Port Dover *[Go to SCREENING 3]*<br>[31] Simcoe *[Go to SCREENING 3]*<br>[32] Tillsonburg *[Go to SCREENING 3]*<br>[33] Waterford *[Go to SCREENING 3]*<br>[ALWAYS DISPLAY]<br>[66] or another town or village? (please specify: ________________________)<br>*[Proceed with Survey, periodically check responses post-survey]*<br>[77] Home is rural (outside a town or village, e.g., rural, agricultural or wilderness area)<br>*[Proceed with Survey]*<br>[-- DO NOT READ THE FOLLOWING ALOUD --]<br>[88] Don't know *[TERMINATE SURVEY]*<br>[99] Decline to Respond *[TERMINATE SURVEY]*<br><br>SCREENING3<br>You appear to live in a community that is excluded from the survey area designated for this research. We are interested in talking to people who live in very small towns, villages or rural locations outside of towns.<br><br>Do you live **within** or **outside** the town limits of [TOWN SELECTED IN SCREENING2]?<br>[1] Within town limits *[TERMINATE SURVEY]*<br>[2] Outside town limits *[Probe and clarify. Go back to previous questions if necessary.]*<br>[88] Don't know [Probe]<br>[99] Decline to Respond *[TERMINATE SURVEY]*<br><br>**TERMINATE SURVEY FOR URBAN RURAL SCREENING.** In order to participate in the survey, you would need to live within the specific rural areas and towns we are interested in. We cannot proceed any further in the survey. Thank you for your time!<br><br>PCODE<br>What is your postal code?<br>| - - - - - - | [allow 6]<br>[88] Don't know/Refused<br>GO TO NEXT BLOCK<br><br>**SCREENING/STATS BLOCK END**
ALL	**RANDOM2 determined the FORM A/B split**<br>**IF RANDOM2 < 50, administered FORM A**<br>**IF RANDOM2 >= 50, administered FORM B**
ALL	**DIARY BLOCK START**<br><br>I would like to ask you about the things [fill name] did yesterday – from midnight [fill pday] night to midnight last night.<br><br>TYPE [1] to begin diary<br><br>THE FOLLOWING INFORMATION IS ASKED FOR EACH ACTIVITY:<br>1. START TIME OF ACTIVITY

	2. DESCRIPTION OF ACTIVITY<br>3. WHERE ACTIVITY WAS DONE<br>4. END TIME OF ACTIVITY
ALL	***1st Activity Entry and Introduction***<br><br>An important part of this study is to learn what kinds of pollutants adult and children come in contact with in their daily activities. To do this we need to find out how and where people spend their time on a wide range of activities, no matter how brief the activity was.<br><br>I would like to ask you about the things [fill name or your child] did yesterday – from midnight [fill pday] to midnight [fill pday].<br><br>An activity is anything [fill name or your child] did during the day. Activities include active tasks such as socializing, preparing food, or eating; more quiet tasks like thinking and relaxing, or traveling such as taking the bus or driving to places. It also includes routine activities such as dressing, personal hygiene, and break at work or school or personal activities such as hugging and kissing. Right now, you are talking to me on the telephone; Talking on the telephone is one type of activity.<br><br>The location can be a specific room inside a home such as the dinning room, bathroom, and bedroom or more general location such as office building, dry cleaning shop, or a farm. In particular, Health Canada is interested in locations where one may be exposed to environmental agents or pollutants such as gas station, garage, and parking lots.<br><br>I would like to remind you that this survey is strictly confidential and your responses will remain anonymous.<br><br>To simplify the process we will begin with midnight on the first day and work forward through the day. What were [fill name or your child] doing at midnight?<br><br>START TIME (24 hour clock): [fill time]<br><br>----- ACTIVITY ------<br><br>[Activity Code List]<br><br>Please use the box below if the activity is not available above OR if there is additional information about the activity to add.<br><br>Activity: ________<br><br>----- LOCATION ------<br><br>And where did this activity take place?<br><br>[Location Code List]<br><br>Please use the box below if the activity is not available above OR if there is additional information about the location to add.<br><br>Location:_______________

	------ END TIME -------<br><br>We have already established that this activity begin before or at midnight, when did it end?<br>If Needed: For example, if [fill name or your child] were sleeping, when did [fill name or your child] wake up? Or if [fill name or your child] was eating a snack in the kitchen, when did [fill name or your child] finish eating the snack?<br><br>TIME (24 hour clock, HHMM):____________<br><br>***Subsequent Activity Entry***<br><br>TIME DIARY<br><br>Continuing with the things [fill name or your child] did yesterday – from midnight [fill pday] to midnight [fill pday].<br><br>[fill name or your child] finished [recall previous activity] at [recall previous time] so what was the next thing [fill name or your child] did?<br><br>START TIME (24 hour clock): [recall previous time]<br><br>----- ACTIVITY ------<br><br>[Activity Code List]<br><br>Please use the box below if the activity is not available above OR if there is additional information about the activity to add.<br><br>Activity: ________<br><br>----- LOCATION ------<br><br>And where did this activity take place?<br><br>[Location Code List]<br><br>Please use the box below if the activity is not available above OR if there is additional information about the location to add.<br><br>Location:_______________<br><br>------ END TIME -------<br><br>And when did this activity end? NOTE: The final activity of the day MUST have an end time equal to 2359.<br><br>Reminder last activity ended at [recall previous time]<br><br>TIME (24 hour clock, HHMM):____________<br>THIS WAS THE LAST ACTIVITY (ended at or after Midnight)

	**Activities/Locations Summary Table Page**<br><br>Activity Start / Activity End / What was the activity? / And where did this activity take place? / (blank)<br>[recall time] / [recall time] / [recall activity] / [recall location] / Edit<br><br>Proceed with next entry<br>OR<br>Entries complete, select continue.
ALL	When location codes 301-310, 313, and 315 are entered, a supplemental question will be asked:<br><br>QT<br>Was this activity conducted on or near a roadway with moderate to heavy traffic?<br><br>(Surveyor Note: A roadway with "moderate to heavy traffic" is one that has a substantial amount of traffic for several hours of the day, such as a main thoroughfare, a busy boulevard, or a highway. It does not include quiet residential streets or roads with occasional traffic.)<br><br>[1] NO<br>[2] YES *[QTa]*<br>[88] DK<br><br>QTA<br>About how much of this activity was conducted on or near a roadway with moderate to heavy traffic?<br>[0] LESS THAN 1 MINUTE<br>[1-120] RECORD THE NUMBER OF MINUTES<br>[180] 3 HOURS<br>[240] 4 HOURS<br>[300] 5 HOURS<br>[360] 6 HOURS<br>[420] 7 HOURS<br>[480] 8 HOURS<br>[540] 9 HOURS<br>[600] 10 HOURS<br>[660] 11 HOURS<br>[720] 12 HOURS<br>[800] MORE THAN 12 HOURS<br>[88] DK<br><br>**DIARY BLOCK END**
ALL	SURVEY CONSIDERED TO BE COMPLETE AFTER COMPLETING THE DIARY AS EARLY TERMINATION
ALL	**TRAFFIC BLOCK START**<br><br>TRAFFIC1<br>Thinking now about [fill NNAM] [fill ap] time spent outdoors yesterday, were there any times when [fill name] [fill were] in a car, van, truck or bus in moderate to heavy traffic?<br>[1] NO *[Traffic2]*

Nested table (Activities/Locations Summary Table Page):

Activity Start	Activity End	What was the activity?	And where did this activity take place?	
[recall time]	[recall time]	[recall activity]	[recall location]	Edit

	[2] YES<br>[88] DK *[Traffic2]*<br><br>TRAFFIC1A<br>For how long altogether?<br>[1] LESS THAN 1 MINUTE<br>[2] RECORD THE NUMBER OF MINUTES<br>[180] 3 HOURS<br>[240] 4 HOURS<br>[300] 5 HOURS<br>[360] 6 HOURS<br>[420] 7 HOURS<br>[480] 8 HOURS<br>[540] 9 HOURS<br>[600] 10 HOURS<br>[660] 11 HOURS<br>[720] 12 HOURS<br>[800] MORE THAN 12 HOURS<br>[88] DK<br><br>TRAFFIC2<br>Were there any times yesterday when [fill name] [fill were] running, walking or standing alongside a road with moderate to heavy traffic?<br>[1] NO *[NEXT BLOCK]*<br>[2] YES<br>[88] DK *[NEXT BLOCK]*<br><br>TRAFFIC2A<br>For how long altogether?<br>[1] LESS THAN 1 MINUTE<br>[2] RECORD THE NUMBER OF MINUTES<br>[180] 3 HOURS<br>[240] 4 HOURS<br>[300] 5 HOURS<br>[360] 6 HOURS<br>[420] 7 HOURS<br>[480] 8 HOURS<br>[540] 9 HOURS<br>[600] 10 HOURS<br>[660] 11 HOURS<br>[720] 12 HOURS<br>[800] MORE THAN 12 HOURS<br>[88] DK<br><br>**TRAFFIC BLOCK END**
ALL	**PARKING BLOCK START**<br><br>PARK1<br>Were there any times yesterday when [fill name] [fill were] in an enclosed parking garage or an indoor parking lot?<br>[1] NO *[Park2]*<br>[2] YES

[88] DK *[Park2]*

PARK1A
For how long altogether?

[1] LESS THAN 1 MINUTE
[2] RECORD THE NUMBER OF MINUTES
[180] 3 HOURS
[240] 4 HOURS
[300] 5 HOURS
[360] 6 HOURS
[420] 7 HOURS
[480] 8 HOURS
[540] 9 HOURS
[600] 10 HOURS
[660] 11 HOURS
[720] 12 HOURS
[800] MORE THAN 12 HOURS
[88] DK

PARK2
Were there any times yesterday when [fill name] [fill were] walking outside to a car in an open or outside parking area?

[1] NO *[NEXT BLOCK]*
[2] YES
[88] DK *[NEXT BLOCK]*

PARK2A
For how long altogether?

[1] LESS THAN 1 MINUTE
[2] RECORD THE NUMBER OF MINUTES
[180] 3 HOURS
[240] 4 HOURS
[300] 5 HOURS
[360] 6 HOURS
[420] 7 HOURS
[480] 8 HOURS
[540] 9 HOURS
[600] 10 HOURS
[660] 11 HOURS
[720] 12 HOURS
[800] MORE THAN 12 HOURS
[88] DK

PARKING BLOCK END

ALL

GAS STATION BLOCK START

GSTAT1
Did [fill name] spend ANY time yesterday at a gas station or auto repair shop?

[1] NO *[NEXT BLOCK]*
[2] YES
[88] DK *[NEXT BLOCK]*

	GSTAT2<br>About how long altogether yesterday did [fill name] spend in those places?<br>[1] LESS THAN 1 MINUTE<br>[2] RECORD THE NUMBER OF MINUTES<br>[180] 3 HOURS<br>[240] 4 HOURS<br>[300] 5 HOURS<br>[360] 6 HOURS<br>[420] 7 HOURS<br>[480] 8 HOURS<br>[540] 9 HOURS<br>[600] 10 HOURS<br>[660] 11 HOURS<br>[720] 12 HOURS<br>[800] MORE THAN 12 HOURS<br>[88] DK<br><br>GSTAT3<br>Did you pump any gasoline yesterday at a gas station?<br>[1] NO<br>[2] YES<br>[88] DK<br><br>GSTAT4<br>[fill were] [fill name] in a car when another person was pumping gasoline?<br>[1] NO<br>[2] YES<br>[88] DK<br>*[NEXT BLOCK]*<br><br>**GAS STATION BLOCK END**
ALL	**ENGINE BLOCK START**<br><br>ENGINE1<br>Yesterday, did any of [fill name] [fill ap] activities at home, at [fill NPLA] or elsewhere involve working with or being near) any gasoline or diesel-powered equipment, besides automobiles?<br>[1] NO<br>[2] YES *[Engine2]*<br>[88] DK<br>*[NEXT BLOCK]*<br><br>NPLA = **work** if ADULT survey<br>NPLA = **school** if CHILD direct or proxy AND KAGE >= 6.<br>NPLA = **day care** if CHILD direct or proxy AND KAGE < 6.<br><br>ENGINE2<br>About how long?<br>[1] LESS THAN 1 MINUTE<br>[2] RECORD THE NUMBER OF MINUTES<br>[180] 3 HOURS<br>[240] 4 HOURS<br>[300] 5 HOURS

	[360] 6 HOURS<br>[420] 7 HOURS<br>[480] 8 HOURS<br>[540] 9 HOURS<br>[600] 10 HOURS<br>[660] 11 HOURS<br>[720] 12 HOURS<br>[800] MORE THAN 12 HOURS<br>[88] DK<br><br>**ENGINE BLOCK END**
FORMA	**WATER BLOCK START**<br><br>WATER1 (ADULT A only)<br>Do you get water for general household use from:<br>[1] THE PUBLIC WATER SYSTEM<br>[2] A PRIVATE WELL<br>[77] OR FROM SOME OTHER SOURCE – WHAT IS THAT? [specify]<br>[88] DK<br><br>WATER2<br>We are also interested in contact [fill name] had with tap water, that is, water from a faucet. First, how many times would you say [fill name] washed [fill your] hands yesterday?<br>[99] NONE<br>[1] 1-2<br>[2] 3-5<br>[3] 6-9<br>[4] 10-19<br>[5] 20-29<br>[6] 30+ TIMES<br>[88] DK<br><br>WATER3<br>Did [fill name] take a shower yesterday?<br>[1] NO *[Water4]*<br>[2] YES<br>[88] DK *[Water4]*<br><br>WATER3A<br>How many showers did [fill name] take?<br>[1] RECORD ACTUAL NUMBER<br>[11] More than 10<br>[88] DK<br><br>WATER3B<br>How long did [fill name] spend taking the shower(s)? (IN TOTAL)<br>[1] RECORD ACTUAL MINUTES<br>[61] OVER AN HOUR<br>[88] DK<br><br>WATER3C<br>When [fill name] showered, was the bathroom door closed?

	[1] NO<br>[2] YES<br>[88] DK<br><br>WATER3D<br>Was there a window open or an exhaust fan on (in the bathroom)?<br>[1] NO<br>[2] YES<br>[88] DK<br><br>WATER4<br>Did you take a bath yesterday or give any children a bath?<br>[1] NO *[NEXT BLOCK]*<br>[2] YES *[Water4a]*<br>[88] DK *[NEXT BLOCK]*<br><br>WATER4A<br>How many baths did [fill name] take (and/or give)?<br>[1] RECORD ACTUAL NUMBER OF BATHS<br>[88] DK<br><br>WATER4B<br>How long did [fill name] spend taking or giving the bath(s)? (IN TOTAL)<br>[1] RECORD ACTUAL MINUTES<br>[61] OVER AN HOUR<br>[88] DK<br><br>WATER4c<br>Was the bathroom door closed?<br>[1] NO<br>[2] YES<br>[88] DK<br><br>WATER4D<br>Was there a window open or an exhaust fan on (in the bathroom)?<br>[1] NO<br>[2] YES<br>[88] DK<br><br>**WATER BLOCK END**
FORMA	**SOLVENT BLOCK START**<br><br>SOLVENT1<br>Yesterday, did any of [fill name] [fill ap] activities at home, at [fill NPLA: school/day care/work] or elsewhere involve working with or being near) Solvents, fumes or strong smelling chemicals?<br>[1] NO<br>[2] YES *[Solvent2]*<br>[88] DK<br>[NEXT BLOCK]<br><br>NPLA = **work** if ADULT survey<br>NPLA = **school** if CHILD direct or proxy AND KAGE >= 6.

	NPLA = **day care** if CHILD direct or proxy AND KAGE < 6.<br><br>SOLVENT2<br>About how long?<br>[1] LESS THAN 1 MINUTE<br>[2] RECORD THE NUMBER OF MINUTES<br>[180] 3 HOURS<br>[240] 4 HOURS<br>[300] 5 HOURS<br>[360] 6 HOURS<br>[420] 7 HOURS<br>[480] 8 HOURS<br>[540] 9 HOURS<br>[600] 10 HOURS<br>[660] 11 HOURS<br>[720] 12 HOURS<br>[800] MORE THAN 12 HOURS<br>[88] DK<br><br>**SOLVENT BLOCK END**
NONPROXY	**SMOKING BLOCK START**<br><br>SMOKE1<br>These next questions are about smoking. Did you smoke any cigarettes yesterday?<br>[1] NO *[Smoke2]*<br>[2] YES *[Smoke1a]*<br>[88] DK *[Smoke2]*<br><br>SMOKE1A<br>About how many cigarettes did you smoke while at home yesterday?<br><br>Surveyor: At home means both indoor and outdoor.<br>[1] NONE<br>[2] 1-2<br>[3] 3-5<br>[4] 6-9<br>[5] 10-14<br>[6] 15-24 (ONE PACK)<br>[7] 25-35<br>[8] 36+ (TWO PACKS OR MORE)<br>[88] DK<br><br>SMOKE1B<br>About how many cigarettes did you smoke away from home yesterday?<br>[1] NONE<br>[2] 1-2<br>[3] 3-5<br>[4] 6-9<br>[5] 10-14<br>[6] 15-24 (ONE PACK)<br>[7] 25-35<br>[8] 36+ (TWO PACKS OR MORE)

	[88] DK<br><br>SMOKE2<br>Did you smoke any cigars or pipe tobacco yesterday?<br>[1] NO *[DNO1]*<br>[2] YES<br>[88] DK *[DNO1]*<br><br>SMOKE2A<br>For about how long?<br>[1] RECORD NUMBER OF MINUTES<br>[60] ONE HOUR<br>[61] MORE THAN 1 HOUR<br>[62] MORE THAN 2 HOURS<br>[63] MORE THAN 3 HOURS<br>[64] MORE THAN 4 HOURS<br>[65] MORE THAN 5 HOURS<br>[66] MORE THAN 6 HOURS<br>[88] DK<br><br>**DNO1**<br>[if Smoke1 ne <1> then goto Smoke3]<br>[if Smoke1a eq <1> then goto Smoke3e]<br>[if Smoke1a eq <8> then goto Smoke3]<br>[goto Smoke3e]
ADULT	SMOKE3<br>Did anybody smoke cigarettes at your home yesterday?<br>[1] NO<br>[2] YES *[Smoke3a]*<br>[88] DK<br>[NEXT BLOCK]<br><br>SMOKE3E<br>Did anybody other than yourself smoke cigarettes at your home yesterday? [equiv Smoke3]<br>[1] NO<br>[2] YES *[Smoke3a]*<br>[88] DK<br><br>SMOKE3A<br>About how many cigarettes altogether did they smoke at your home yesterday?<br>[1] NONE<br>[2] 1-2<br>[3] 3-5<br>[4] 6-9<br>[5] 10-14<br>[6] 15-24 (ONE PACK)<br>[7] 25-35<br>[8] 36+ (TWO PACKS OR MORE)<br>[88] DK<br><br>**SMOKING BLOCK END**

CHILD	**CHILD SMOKING BLOCK START**<br><br>PARENT<br>Now I need to talk to your [fill krel] again. Is [fill krel] available?<br>[1] NO – ask for call back time SAME DAY<br>[2] YES *[SMOKING BLOCK –parent CD version]*<br>[88] REF (will terminate the survey)<br><br>This interview is completely voluntary. If we come to any question you don't want to answer, just tell me and we'll skip it<br><br>PARSMOKE1<br>These next questions are about smoking. Did you or any members of your family or visitors smoke cigarettes in your home yesterday?<br><br>Surveyor: At home means both indoor and outdoor.<br><br>[1] NO *[ParSmoke3]*<br>[2] YES<br>[88] DK *[ParSmoke3]*<br><br>PARSMOKE2<br>About how many cigarettes altogether did you and they smoke at your home yesterday?<br><br>Surveyor: At home means both indoor and outdoor.<br><br>[1] 1-2<br>[2] 3-5<br>[3] 6-9<br>[4] 10-14<br>[5] 15-24 (ONE PACK)<br>[6] 25-35<br>[7] 36+ (TWO PACKS OR MORE)<br>[88] DK<br><br>PARSMOKE3<br>Is smoking allowed in your home?<br>[1] NO<br>[2] YES<br>[88] DK<br><br>**CHILD SMOKING BLOCK END**
ALL	**HOUSE BLOCK 1 START**<br><br>BLOCK1<br>Is your home located on or within 1 block of a roadway with moderate to heavy traffic?<br>[1] NO<br>[2] YES<br>[88] DK
CHILD	BLOCK1A<br><DISPLAY IF SVTYPE GE 3>

	[Asked in Child Direct and Child Proxy Survey, both Form A and B]<br><br>Is the school/daycare [fill name-child] attends located on or within 1 block of a roadway with moderate to heavy traffic?<br>[1] NO<br>[2] Child does not attend school or daycare<br>[3] YES<br>[88] DK
FORMB	BLOCK2<br>Do you get water for general household use from:<br>[1] THE PUBLIC WATER SYSTEM<br>[2] A PRIVATE WELL<br>[77] OR FROM SOME OTHER SOURCE – WHAT IS THAT? [specify]<br>[88] DK
ALL	BLOCK3<br>Do you have an outdoor wood boiler?<br><br>Surveyor: An outdoor wood boiler also known as an outdoor wood furnace is a heating appliance that sits outside the home while providing heat to the home (see What IS Document for more information).<br><br>[1] NO<br>[2] YES<br>[88] DK<br><br>**HOUSE BLOCK 1 END**
ALL	**GARAGE BLOCK START**<br><br>GARAGE1<br>Do you live in a house with an attached garage?<br><br>Surveyor: An apartment with underground parking garage does not qualify. A house, townhouse, or duplex qualifies.<br>[1] NO<br>[2] YES *[Garage2]*<br>[88] DK<br>*[NEXT BLOCK]*<br><br>GARAGE2<br>Is there a door connecting the garage to the home?<br>[1] NO *[Garage3]*<br>[2] YES *[Garage3]*<br>[88] DK *[Garage3]*<br><br>GARAGE3<br>Yesterday, was any automobile or other motor vehicle started in the garage?<br>[1] NO<br>[2] YES *[Garage4]*<br>[88] DK<br>*[NEXT BLOCK]*

	GARAGE4<br>How many times? Would you say: (read list)<br>[1] 1-2<br>[2] 3-5<br>[3] 6-9<br>[4] OR 10 OR MORE<br>[88] DK<br>*[NEXT BLOCK]*<br><br>GARAGE5<br>About how many of these times were with the garage doors closed? Would you say:<br><br>SURVEYOR: This means the large garage doors to outside, not a door into the house. (read list)<br>[1] None<br>[2] 1-2<br>[3] 3-5<br>[4] 6-9<br>[5] OR 10 OR MORE<br>[88] DK<br>*[NEXT BLOCK]*<br><br>GOWI<br>I recorded that the car was started with the garage door closed more than the total number of times the car was started. Let me go back over these questions one more time.<br>TYPE [g] TO RETURN TO QUESTION Garage4 *[Garage4]*<br><br>**GARAGE BLOCK END**
FORMA	**STORAGE BLOCK START**<br><br>STORE1<br>Is any gasoline or kerosene being stored in any room or basement of your home or in an attached garage or carport?<br>[1] NO<br>[2] YES<br>[88] DK<br><br>STORE2<br>Are any devices with gasoline engines such as lawn mowers being stored in any room or basement of your home or in an attached garage or carport?<br>[1] NO<br>[2] YES<br>[88] DK<br><br>STORE3<br>Are any paints or varnishes being stored in any room or basement of your home or in an attached garage or carport?<br>[1] NO<br>[2] YES<br>[88] DK<br><br>STORE4

	Are any mothballs, moth crystals or moth cakes used in your home?<br><br>Surveyor: Mothballs or other moth repellent are products containing chemical pesticide and deodorant used when storing clothing and other articles susceptible to damage from mold or moth larvae.<br><br>A moth is an insect related to butterfly and is commonly regarded as pests because their larvae eat fabric such as clothes and blankets made from natural fibers such as wool or silk.<br><br>[1] NO<br>[2] YES<br>[88] DK<br><br>**FORM A -STORAGE BLOCK END**
ALL	**AIR CONDITION BLOCK START**<br>**ASKED IN SUMMER ONLY**<br><br>AC1<br>Is your home air-conditioned?<br>[1] NO<br>[2] YES *[AC2]*<br>[88] DK<br>*[NEXT BLOCK]*<br><br>AC2<br>Do you use a central unit or a window or wall unit?<br>[1] CENTRAL<br>[2] WINDOW/WALL<br>[88] DK<br><br>AC3<br>Was the air conditioning on at any time yesterday?<br>[1] NO<br>[2] YES *[AC4]*<br>[88] DK<br><br>AC4<br>Did [fill name] spend anytime at home yesterday while the air conditioning was on?<br>[1] NO<br>[2] YES<br>[88] DK<br><br>**AIR CONDITIONING BLOCK END**
ALL	**HEATING BLOCK START**<br>**ASKED IN WINTER ONLY**<br><br>HEAT1<br>Was any heat turned on at any time in your home yesterday?<br>[1] NO *[NEXT BLOCK]*<br>[2] YES *[Heat2]*<br>[88] DK *[NEXT BLOCK]*

	HEAT2<br>Was a central furnace turned on?<br>[1] NO *[Heat5]*<br>[2] YES<br>[88] DK *[Heat5]*<br><br>HEAT3<br>What kind of fuel was used in a central furnace? (FIRST MENTION)<br>[1] GAS<br>[2] ELECTRICITY<br>[3] OIL<br>[4] COAL<br>[5] KEROSENE<br>[6] WOOD<br>[7] SOLAR<br>[77] OTHER (SPECIFY) [specify]<br>[88] DK *[Heat5]*<br><br>HEAT4<br>Any others? (SECOND MENTION)<br>[99] NO OTHERS/NONE<br>[1] GAS<br>[2] ELECTRICITY<br>[3] OIL<br>[4] COAL<br>[5] KEROSENE<br>[6] WOOD<br>[7] SOLAR<br>[77] OTHER (SPECIFY) [specify]<br>[88] DK<br><br>HEAT5<br>Was any room [fill also] heated with a wood stove?<br>[1] NO<br>[2] YES<br>[88] DK<br>*[Heat6]*<br><br>HEAT6<br>Was any room [fill also] heated with a) Kerosene space heater?<br>[1] NO<br>[2] YES<br>[88] DK<br>*[Heat7]*<br><br>HEAT7<br>Was any room [fill also] heated with a) Electric space heater?<br>[1] NO<br>[2] YES<br>[88] DK<br>*[Heat8]*<br><br>HEAT8

	Was any room [fill also] heated with a) Fireplace?<br>[1] NO<br>[2] YES<br>[88] DK<br>*[Heat9]*<br><br>HEAT9<br>Was any room [fill also] heated with a) Any other heat source?<br>[1] NO<br>[2] YES - What was that? [specify]<br>[88] DK<br><br>**HEATING BLOCK END**
FORMB	**HOUSE BLOCK 2 START**<br><br>Now, I have a few more questions about your home.<br><br>HOUSE 1<br>In what year did you move into your home?<br>[XXXX] RECORD ACTUAL YEAR<br>[88] DK/DON'T REMEMBER *[HOUSE2]*<br><br>[AOWI]<br>I recorded that you moved into your home before you were born. You said that you were born in [fill Rage].<br><br>Which of these answers have I recorded wrong?<br>[1] Year moved is wrong, return to previous question<br>[2] Year of birth is wrong. Correct Year __________
ALL	[If location of survey is Annapolis Valley or Haldimand-Norfolk, GO TO Farm. If not, GO TO House2.]<br>FARM<br>Do you live on a farm?<br>[1] NO<br>[2] YES<br>[88] DK<br><br>HOUSE2<br>Now, I have a few more questions about your home. Do you live in an:<br>[1] APARTMENT<br>[2] DETACHED SINGLE FAMILY HOUSE<br>[3] A TOWNHOUSE<br>[4] OR SOMETHING ELSE (SPECIFY) [specify]<br>[88] DK<br><br>HOUSE3<br>Was this building built:<br>[1] since 2001<br>[2] between 1991 and 2000<br>[3] between 1981 and 1990<br>[4] between 1971 and 1980

	[5] between 1951 and 1970<br>[6] or earlier than 1951<br>[88] DK<br><br>HOUSE4<br>How many stories or floors are in your building or home?<br>(COUNT ONLY FLOORS WITH FINISHED ROOMS FOR LIVING PURPOSES OR FULL BASEMENTS)<br><br>A full or finished basement function as a habitable living space in the house. Typical examples include furnished recreation room, living room or a secondary suite.<br><br>An unfinished or partially finished basement serves primarily as a storage space. A basement with only a furnace, water tank, freezer, washer and dryer sets, or other storage would be considered an unfinished basement or partially finished basement.<br><br>[1] ONE<br>[2] TWO<br>[3] THREE<br>[4] 4 to 6<br>[5] 7 to 12<br>[6] 13 OR MORE STORIES<br>[88] DK
FORMB	HOUSE5<br>How many rooms (not counting bathrooms or half-rooms) do you have in your home?<br>[1] RECORD NUMBER<br>[31] MORE THAN 30<br>[88] DK *[House7]*<br><br>HOUSE6<br>Of these rooms, how many are carpeted or have rugs covering most of their surface?<br>** SHOULD BE LESS THAN OR EQUAL TO TOTAL ROOMS: [fill House5]<br>[1] NONE<br>[2] RECORD NUMBER<br>[31] MORE THAN 30<br>[88] DK *[House7]*<br><br>[cowi] [if House5 eq [88] Then goto House7] [if House5 ge House6 then goto House7]<br><br>[COWI]<br>I recorded that you have more carpeted rooms than total rooms. Let me go back over these questions one more time.<br><br>** TYPE [g] TO RETURN TO HOUSE5<br><br>HOUSE7<br>Is there a basement in your building or house?<br>[1] NO<br>[2] YES<br>[88] DK<br><br>**HOUSE BLOCK 2 END**

FORMB	**RENOVATIONS BLOCK START**<br><br>RENO1<br>In the last six months, have you or anyone else renovated your home in any way? This would include indoor painting, refinishing floors, adding rooms to the house or laying new carpet.<br>[1] NO<br>[2] YES *[Reno2]*<br>[88] DK<br>*[NEXT BLOCK]*<br><br>RENO2<br>Was any indoor painting done?<br>[1] NO<br>[2] YES<br>[88] DK<br><br>RENO3<br>How about refinishing floors?<br>[1] NO<br>[2] YES<br>[88] DK<br><br>RENO4<br>What about additions to the house?<br>[1] NO<br>[2] YES<br>[88] DK<br><br>RENO5<br>What about laying carpet?<br>[1] NO *[NEXT BLOCK]*<br>[2] YES *[Reno6]*<br>[88] DK *[NEXT BLOCK]*<br><br>RENO6<br>Was glue used or was it tacked down?<br>[1] GLUE<br>[2] TACKED<br>[77] OTHER<br>[88] DK<br><br>**RENOVATIONS BLOCK END**
FORMB	**FRESH AIR BLOCK START**<br><br>FRESH1<br>Does your home have a Heat Recovery Ventilator (HRV) unit?<br>[1] NO<br>[2] YES<br>[88] DK<br><br>FRESH2<br>Are exhaust fans used in the kitchen?

	[1] NO<br>[2] YES<br>[88] DK<br><br>FRESH3<br>Are exhaust fans used in the bathroom?<br>[1] NO<br>[2] YES<br>[88] DK<br><br>FRESH4<br>Are exhaust fans used in the laundry?<br>[1] NO<br>[2] YES<br>[88] DK<br><br>FRESH5<br>Are open doors and windows used to provide fresh air to your home?<br>[1] NO<br>[2] YES<br>[88] DK<br><br>**FRESH AIR BLOCK END**
FORMB	**WINDOW AND DOOR BLOCK START**<br><br>WINDOW1<br>While [fill name] [fill were] at home yesterday, were any windows left open at all?<br>[1] NO<br>[2] YES *[Window2]*<br>[88] DK<br>*[Door1]*<br><br>WINDOW2<br>How many windows?<br>[1] 1-2<br>[2] 3-5<br>[3] 6-9<br>[4] 10+<br>[88] DK *[Door1]*<br><br>WINDOW3<br>For about how long altogether were any windows left open?<br>[1] LESS THAN 1 MINUTE<br>[2] RECORD ACTUAL NUMBER OF MINUTES (LESS THAN 2 HOURS)<br>[180] FROM 2 TO 4 HOURS<br>[360] MORE THAN 4 UP TO 8 HOURS<br>[600] MORE THAN 8 UP TO 12 HOURS<br>[840] MORE THAN 12 UP TO 16 HOURS<br>[960] OVER 16 HOURS<br>[88] DK<br><br>DOOR1

	Does your home have any doors which open directly to the outside of your building or house?<br>[1] NO *[NEXT BLOCK]*<br>[2] YES<br>[88] DK *[NEXT BLOCK]*<br><br>DOOR2<br>While [fill name] [fill were] at home yesterday, were any of these outside doors left open at all?<br>[1] NO<br>[2] YES *[Door3]*<br>[88] DK<br>*[NEXT BLOCK]*<br><br>DOOR3<br>For about how long altogether was any outside door left open?<br>[1] LESS THAN 1 MINUTE<br>[2] RECORD ACTUAL NUMBER OF MINUTES (LESS THAN 2 HOURS)<br>[180] 2 HOURS TO 4 HOURS<br>[360] MORE THAN 4 UP TO 8 HOURS<br>[480] MORE THAN 8 UP TO 12 HOURS<br>[720] OVER 12 HOURS<br>[88] DK<br><br>DOOR4<br>While [fill name] [fill were] at home yesterday, about how many times would you say that an outside door was opened? Would you say:<br>[1] ONCE OR TWICE<br>[2] 3-5 TIMES<br>[3] 6-9 TIMES<br>[4] 10-19 TIMES<br>[5] OR 20 TIMES OR MORE<br>[88] DK<br><br>**WINDOW AND DOOR BLOCK END**
FORMB	**GAS OVEN BLOCK START**<br><br>OVEN1<br>Do you have a gas range or gas oven in your home?<br>[1] NO<br>[2] YES *[Oven2]*<br>[88] DK<br>*[NEXT BLOCK]*<br><br>OVEN2<br>Does your gas range or gas oven have a continuously burning pilot light?<br>[1] NO<br>[2] YES<br>[88] DK<br><br>OVEN3<br>Did [fill name] spend any time yesterday at home when the gas range or gas oven was used?<br>[1] NO<br>[2] YES *[Oven4]*

	[88] DK<br>*[NEXT BLOCK]*<br><br>OVEN4<br>For about how long [fill were] [fill name] home when the range or oven was on?<br>[1] LESS THAN 1 MINUTE<br>[2] RECORD THE NUMBER OF MINUTES<br>[180] 3 HOURS — [480] 8 HOURS<br>[240] 4 HOURS — [540] 9 HOURS<br>[300] 5 HOURS — [600] 10 HOURS<br>[360] 6 HOURS — [660] 11 HOURS<br>[420] 7 HOURS — [720] 12 HOURS<br>[800] MORE THAN 12 HOURS<br>[88] DK<br><br>**GAS OVEN BLOCK END**
FORMA	**PESTICIDE BLOCK START**<br><br>PEST1<br>In the last six months, were any pesticides used to get rid of insects, rodents or other pests at your home?<br><br>Surveyor: Agricultural use of pesticides qualifies as yes.<br>[1] NO<br>[2] YES *[Pest2]*<br>[88] DK<br>*[NEXT BLOCK]*<br><br>PEST2<br>Was that done: (read list)<br><br>[1] INSIDE<br>[2] OUTSIDE<br>[3] OR BOTH?<br>[88] DK<br><br>PEST3<br>How many of these times were pesticides applied by a PROFESSIONAL?<br>[1] NONE<br>[2] 1-2<br>[3] 3-5<br>[4] 6-9<br>[5] 10+ TIMES<br>[88] DK<br><br>PEST4<br>How many of these times did you PERSONALLY apply pesticides?<br>[1] NONE<br>[2] 1-2<br>[3] 3-5<br>[4] 6-9<br>[5] 10+ TIMES

<table>
<tr><td></td><td>[88] DK

PESTICIDE BLOCK END</td></tr>
<tr><td>ALL</td><td>CHILD PLAY BLOCK START

If response to Qkid is 0, 6-17 or 99, go to NEXT BLOCK. If response to Qkid is 1-5, go to Play1.

DIRT
I recorded that you have a young child living in this household. What is the first name of this child?

(Surveyor: this is the child whose age was given as [Qkid or Qkid2].)

[1]**RECORD FIRST NAME** [store [this child] in DIRT] [Play1]
[99]**IF REFUSES TO GIVE NAME TYPE (r) [Play1]

DIRT [store name in dirt]

PLAY1
Yesterday, did [fill dirt] spend any time crawling, lying, or playing on the floor in your home?
[1] NO
[2] YES [Play1a]
[88] DK
[Play2]

PLAY1A
Thinking of that floor area where [fill dirt] spent most of his or her time yesterday, is that area carpeted?
[1] NO, NOT CARPETED
[2] YES CARPETED
[88] DK

PLAY2
Did [fill dirt] spend any time yesterday playing outdoors?
[1] NO
[2] YES [Play3]
[88] DK
[99] REFUSED
[NEXT BLOCK]

PLAY3
When [fill dirt] was outdoors yesterday, did he/she spend any time playing on dirt?
[1] NO
[2] YES [Play3a]
[88] DK
[NEXT BLOCK]

PLAY3A
About how much time was on dirt?
[1] LESS THAN 1 MINUTE
[2] RECORD THE NUMBER OF MINUTES
[180] 3 HOURS</td></tr>
</table>

	[240] 4 HOURS<br>[300] 5 HOURS<br>[360] 6 HOURS<br>[420] 7 HOURS<br>[480] 8 HOURS<br>[540] 9 HOURS<br>[600] 10 HOURS<br>[660] 11 HOURS<br>[720] 12 HOURS<br>[800] MORE THAN 12 HOURS<br>[88] DK<br><br>**CHILD PLAY BLOCK END**
ADULT	**EMPLOYMENT BLOCK START**<br><br>JOB1<br>Now, I have some background questions: Are you currently:<br>[1] EMPLOYED FULL TIME *[Job2]*<br>[2] EMPLOYED PART TIME *[Job2]*<br>[3] OR NOT EMPLOYED AT ALL? *[Job1a]*<br>[99] REF<br><br>JOB1A<br>Is that mainly because you are:<br>[1] RETIRED<br>[2] KEEPING HOUSE<br>[3] TEMPORARILY UNEMPLOYED<br>[4] A STUDENT<br>[5] DISABLED<br>[6] OR SOMETHING ELSE: (SPECIFY) [specify]<br>[99] REF<br><br>JOB2<br>About how many hours last week did you work for pay on this or other jobs?<br>[1] NONE - Why not: (SPECIFY) [specify] *[Job5]*<br>[2] RECORD NUMBER OF HOURS<br>[81] MORE THAN 80 HOURS<br>[88] DK *[Job5]*<br><br>JOB3<br>How many of the work hours last week did you work outdoors? (that is, not inside any building)<br>[1] NONE *[Job4]*<br>[2] RECORD NUMBER OF HOURS<br>[81] MORE THAN 80 HOURS<br>[88] DK *[Job4]*<br><br>JOB4<br>About how many of the work hours last week were between 6 PM in the evening and 6 AM in the morning?<br>[1] NONE *[Job5]*<br>[2] RECORD NUMBER OF HOURS<br>[81] MORE THAN 80 HOURS

	[88] DK [Job5]<br><br>tmow [if Job3 gt Job2 then goto TMOW] *[Job4]*<br>tmmh [if Job4 gt Job2 then goto TMMH] *[Job5]*<br><br>TMOW<br>I recorded that you worked more hours last week outdoors than you worked in total. Let me go back over these questions one more time.<br>TYPE [y] TO RETURN TO QUESTION Job3 *[Job3]*<br><br>TMMH<br>I recorded that you worked more hours last week between 6 PM in the evening and 6 AM in the morning than you worked in total. Let me go back over these questions one more time.<br>TYPE [x] TO RETURN TO QUESTION Job4 *[Job4]*<br><br>JOB5<br>What is the postal code at your (main) place of work? [allow 6, minimum 3] | - - - - - - | <br>JOB6<br>In what kind of business, industry, or service do you work? Please give details<br><br>For example,<br>- new home construction<br>- primary school<br>- municipal police<br>- wheat farm<br>- shoe store<br>- food wholesale<br>- car parts factory<br><br>JOB7<br>What is your work or occupation? Please be specific.<br><br>For example,<br>- legal secretary<br>- plumber<br>- fishing guide<br>- wood furniture assembler<br>- secondary school teacher<br>- (If in the Armed Forces, give rank.)<br><br>JOB8<br>What are your main activities or duties? Please give details:<br>Surveyor: Record at least 3 duties.<br><br>For example:<br>- prepared legal documents<br>- installed residential plumbing<br>- guided fishing parties<br>- made wood furniture products<br><br>**EMPLOYMENT BLOCK END**

ALL	**HEALTH BLOCK START**<br><br>HEALTH1<br>Next, I have a few questions about [fill your] health. Has a doctor told you that [fill name] [fill have] asthma?<br>[1] NO<br>[2] YES<br>[88] DK<br><br>HEALTH2<br>Has a doctor told you that [fill name] [fill have] heart disease?<br>[1] NO<br>[2] YES<br>[88] DK<br><br>HEALTH3<br>Has a doctor told you that [fill name] [fill have] Chronic bronchitis or emphysema?<br>[1] NO<br>[2] YES<br>[88] DK<br><br>HEALTH4<br>Does a long-term physical condition, mental condition, or health problem, reduce the amount or kind of activity [fill name] can do? A “long-term condition” refers to a condition that is expected to last or has already lasted 6 months or more.<br>[1] SOMETIMES<br>[2] OFTEN<br>[3] NEVER<br>[88] DK<br>[99] REF<br><br>**HEALTH BLOCK END**
ADULT	**EDUCATION BLOCK START**<br><br>EDUC1<br>What is the last grade or year of school you completed?<br><br>[1] NONE<br>[2] SOME ELEMENTARY<br>[8] ELEMENTARY GRADUATE<br>[10] SOME HIGH SCHOOL<br>[12] HIGH SCHOOL GRADUATE<br>[14] SOME COLLEGE OR CEGEP<br>[16] COLLEGE OR CEGEP GRADUATE<br>[17] SOME UNIVERSITY<br>[18] UNIVERSITY GRADUATE<br>[19] SOME GRADUATE SCHOOL<br>[20] GRADUATE OR PROFESSIONAL DEGREE<br>[99] REFUSED<br><br>**EDUCATION BLOCK END**
ALL	**INCOME BLOCK START**

	INCQ1<br>I now have a question regarding your household income. Please be assured that, like all other information you have provided, these answers will be kept strictly confidential. What is your best estimate of the total income, before taxes and deductions, of all household members from all sources in the past 12 months?<br>[allow 7] [MIN: 0] [MAX: 1,000,000; warning after 250,000, go to QINCQ1TEST]<br>[1] | - - - - - - | [2] $0 *[VERF]*<br>[3] MORE THAN $500,000 *[VERF]*<br>[88] DK/REF *[INCQ2]*<br><br>QINCQ1TEST<br>You have said that your household income is [$]. Is this correct?<br><br>INCQ2<br>Can you estimate in which of the following groups your household income falls?<br><br>[1] $0 to $5,000 *[VERF]*<br>[2] $5,001 to $10,000 *[VERF]*<br>[3] $10,001 to $15,000 *[VERF]*<br>[4] $15,001 to $20,000 *[VERF]*<br>[5] $20,001 to $30,000 *[VERF]*<br>[6] $30,001 to $40,000 *[VERF]*<br>[7] $40,001 to $50,000 *[VERF]*<br>[8] $50,001 to $60,000 *[VERF]*<br>[9] $60,001 to $80,000 *[VERF]*<br>[10] $80,001 to $100,000 *[VERF]*<br>[11] $100,001 or more *[VERF]*<br>[88] DK *[VERF]*<br>[99] RF *[VERF]*<br><br>**INCOME BLOCK END**
ALL	**VERIFICATION BLOCK START**<br><br>VERF<br>And, finally, your number is [fill racd] [fill tel1]-[fill tel2:0]?<br><br>[1] NO - What number have I reached? (SPECIFY) [specify]<br>[2] YES<br>[99] REF<br><br>**VERIFICATION BLOCK END**
ALL	**DRAW BLOCK START**<br><br>DRAW1<br>By completing this survey, you are eligible for entry in a draw to win a cash prize valued at $300. Would you like to enter into the prize draw?<br><br>[1] YES *[CONTACT1]*<br>[2] NO *[END]*

	CONTACT1<br>Please provide your first name and the best telephone number(s) to reach you should you be randomly selected as the winner.<br><br>Any information you provide will be stored for the purpose of the draw only. Your name and phone number will not be associated with your survey responses. All contact information will be destroyed after the draw has taken place and winners notified.<br><br>[1] First Name ______________________________ *[END]*<br>[2] Home Phone______________________________ *[END]*<br>[3] Cell Phone_______________________________ *[END]*<br>[4] Other Phone______________________________ *[END]*<br><br>**DRAW BLOCK END**

Infant Survey

	**CHAPS 2 - INFANT COMBINED FORM A & B QUESTIONNAIRE**<br><br>**ALL – Applicable to all -infant survey form A and B**<br>**FORMA – Applicable to all survey type but only form A**<br>**FORMB – Applicable to all survey type but only form B**<br><br>**Code / Survey Type / Form**<br>**ALL / Infant / A & B**<br>**FORMA / Infant / A**<br>**FORMB / Infant / B**
ALL	INTRODUCTION BLOCK START<br><br>INTRO1<br><br>**For all locations other than Montreal:**<br>Hello, my name is ____. I'm calling for Health Canada.<br><br>Bonjour, je m'appelle _____. J'appelle de la part de Santé Canada.<br><br>Would you prefer to continue in English or French?<br><br>**For Montreal:**<br>Bonjour, je m'appelle _____. J'appelle de la part de Santé Canada.<br><br>Hello, my name is ____. I'm calling for Health Canada.<br><br>Préférez-vous continuer en anglais ou en français?<br><br>*Remainder to be conducted in language of choice.*<br><br>** IF YOUNG CHILD ANSWERS, ASK TO SPEAK WITH AN ADULT<br><br>We are conducting a study to assess Canadian's exposure to pollutants. Your responses will inform Health Canada on how to protect the health of Canadians.<br><br>If you complete the survey, you can enter into a draw to win a cash prize of $300. The draws will take place on September 30, 2010 (*summer*) March 31, 2011 (*winter*).<br><br>[Surveyor: If asked about odds of winning, read] Your chance of winning is approximately 1 in 75.<br><br>Would you be able to do the survey with me?<br><br>If no, read<br><br>While participation is voluntary, your cooperation is important to ensure the information collected is as accurate as possible. Your participation is also important because your response will represent not only your household, but similar households in your area.<br><br>Would you like to take part in this study?<br><br>If yes, continue:

Thank you. Before we begin, would you have time for me to ask you a few questions to determine which person in your household should complete the survey?

[Surveyor: if asked, read] The survey selects one respondent depending on who lives in your household and who next celebrate his or her birthday.
[Surveyor: If asked about survey length, read]
The survey can be completed in 20 minutes or less.

[If pressed, read] Depending on how you answer each question, it may take you more or less time to go through the survey.

FIRST, are there any infants under 1 year living in this household? [loc 1/34]

[1] NO (TERMINATE SURVEY)
[2]YES (BEGIN SURVEY) *[Qnadult]*
[3] Not now – ask for call-back time
[99]Refused (will terminate survey) *[Thanks and Terminate]*

Thank you. The study will update information collected 15 years ago. The survey asks you to recall your infant activities from the previous day and asks specific questions relating to your home. Knowing where and how Canadians spend their time will help Health Canada to estimate Canadians' exposure to pollutants. These estimates help set standards to protect the health of Canadians.

Participation is voluntary and you can skip over questions you do not want to answer. Your name and other identifying information will not be associated with your responses and any information you provided will be used for research purposes only and will be kept strictly confidential.

KNAM
For this study, we need to know about the activities of the infant in the household under the age of 1 year who will have the NEXT birthday. Who would that be? I just need his or her first name.

[1] RECORD FIRST NAME BELOW *[kage]*
[99] IF REFUSES TO GIVE NAME TYPE (r) *[kage]*

KAGE
How old is [fill NAME]?

[1] LESS THAN 6 MONTHS *[ksex]*
[2] 6 TO 11 MONTHS *[ksex]*
[99] REF/DK *[ksex]*

KSEX
** IF NOT SURE ASK: Is [fill NAME] a boy or girl?

[1] BOY
[2] GIRL
[99] REF

WKID
I will need to speak with an adult, who lives in the household who would know how [fill NAME-child] spent [fill YOUR] time yesterday. Who would that be?

[1] INFORMANT *[krel]*
[2] SOMEONE ELSE *[home]*
[99] REFUSED (will terminate survey) *[RES1]*

HOME
During this interview I will need to have [fill know] available to complete the survey. Is [fill know] home now?

SURVEYOR: Introduction for new person, if necessary:
We are conducting a study to help reduce the exposure of both adults and children to pollution. Your answers are for statistical purposes only, and will be kept strictly confidential. This interview is completely voluntary. If we come to any question you don't want to answer, just tell me and we'll skip it.
May I start the survey with you?

[1] NO *[Thanks and Terminate]*
[2] YES(Yes I now need to speak to that person, please) *[krel]*
[99] REF *[Thanks and Terminate]*

**if home = 1, say: I now need to speak with [fill know-adult], please. *[KREL]*

KREL
What is your relationship to [fill NAME]?
[1] MOTHER
[2] FATHER
[3] OTHER - SPECIFY: [specify]
[99] REFUSED

INTRODUCTION BLOCK END

ALL

SCREENING/STATS BLOCK START

Before asking about what [fill NAME] did yesterday, I need to ask you a few questions about the composition of your household.

QNADULT
Including yourself, how many adults, age 18 and older, live in your household?
[1] RECORD ACTUAL NUMBER *[nkid1]*
[11] MORE THAN 10 *[nkid1]*
[99] REF *[nkid1]*

NKID1
How many children under 18 live in your household?
[2] ONE *[Qkid2]*
[3] RECORD NUMBER *[Qkid2]*
[11] MORE THAN 10 *[Qkid2]*
[99] REF *[Qkid2]*

QKID2
Aside from your infant(s), what is the age of the youngest child living in your household?
[0] ONLY INFANT *[Qninfant]*
[2] RECORD AGE 1 THRU 17 *[RANDOM1]*
[99] DK/REF *[RANDOM1]*

	[loc 3/62]<br><br>QNINFANT<br>Just to confirm, how many infants under 1 year live in your household?<br>[1] RECORD NUMBER *[RANDOM1]*<br>[99] REF *[RANDOM1]*<br><br>Phone1<br>All together, how many different landline phone NUMBERS does your household have for non-business use?<br><br>SURVEYOR: If respondent does not know what a landline is, read: A land line is a regular telephone line, typically served over a pair of copper wires from a phone company into your home in a fixed location. They are named as such because they are connected to a physical line rather than a cell tower or satellite. It is not a cell phone or mobile phone.<br><br>[1] No landlines<br>[2] RECORD ACTUAL NUMBER (1-5)<br>[6] 6 OR MORE<br>[99] REF<br><br>Phone2<br>All together, how many different cell phone NUMBERS does your household have for non-business use?<br>[1] No landlines<br>[2] RECORD ACTUAL NUMBER (1-5)<br>[6] 6 OR MORE<br>[99] REF<br><br>**[ASK SCREENING BLOCK ONLY IF REGION=6 {Annapolis Valley} OR REGION=7 {Haldimand Norfolk}]**<br>SCREENING1<br>What county is your home located in? [If necessary, read county list]<br>[ALTERNATE WORDING IF ONLY ONE COUNTY: Is your home located within...? READ COUNTY NAME]<br><br>[DISPLAY OPTIONS IF REGION=6]<br>[11] Annapolis County *[Go to SCREENING 2]*<br>[12] Kings County *[Go to SCREENING 2]*<br>[DISPLAY OPTIONS IF REGION=7]<br>[31] Haldimand County *[Go to SCREENING 2]*<br>[32] Norfolk County *[Go to SCREENING 2]*<br>[ALWAYS DISPLAY]<br>[77] Another county (please specify): ______________ *[TERMINATE SURVEY]*<br>[88] Don't Know *[Go to SCREENING 2]*<br>[99] Decline to Respond *[TERMINATE SURVEY]*<br><br>[If selected valid County in SCREENING1, list 'exclusion towns' within the sample region, as well as a few towns just outside the boundaries of the County that are excluded as well]<br><br>SCREENING2<br>Is your home located in a rural area? or <u>within</u> one of the following towns? [Read list]<br>[DISPLAY OPTIONS IF REGION=6]

	[11] Bridgetown	*[Go to SCREENING 3]*
	[12] Middleton	*[Go to SCREENING 3]*
	[13] Digby	*[Go to SCREENING 3]*
	[14] Kingston	*[Go to SCREENING 3]*
	[15] Greenwood	*[Go to SCREENING 3]*
	[16] Berwick	*[Go to SCREENING 3]*
	[17] Coldbrook	*[Go to SCREENING 3]*
	[18] Kentville	*[Go to SCREENING 3]*
	[19] Hantsport	*[Go to SCREENING 3]*
	[111] Annapolis	*[Go to PCODE]*
	[112] Annapolis Royal	*[Go to PCODE]*
	[113] Aylesford	*[Go to PCODE]*
	[114] Canning	*[Go to PCODE]*
	[115] Cornwallis	*[Go to PCODE]*
	[116] Deep Brook	*[Go to PCODE]*
	[117] Granville Ferry	*[Go to PCODE]*
	[118] Hampton	*[Go to PCODE]*
	[119] Kingsport	*[Go to PCODE]*
	[120] Lawrencetown	*[Go to PCODE]*
	[121] Margaretsville	*[Go to PCODE]*
	[122] Melvern Square	*[Go to PCODE]*
	[123] Morden	*[Go to PCODE]*
	[124] Mt Rose	*[Go to PCODE]*
	[125] Nictaux	*[Go to PCODE]*
	[126] Paradise	*[Go to PCODE]*
	[127] Spa Springs	*[Go to PCODE]*
	[128] Wilmot	*[Go to PCODE]*
	[129] Youngs Cave	*[Go to PCODE]*
	[DISPLAY OPTIONS IF REGION=7]	
	[20] Windsor	*[Go to SCREENING 3]*
	[21] Brantford	*[Go to SCREENING 3]*
	[22] Burtford	*[Go to SCREENING 3]*
	[23] Caledonia	*[Go to SCREENING 3]*
	[24] Cayuga	*[Go to SCREENING 3]*
	[25] Delhi	*[Go to SCREENING 3]*
	[26] Dunnville	*[Go to SCREENING 3]*
	[27] Hagersville	*[Go to SCREENING 3]*
	[28] Jarvis	*[Go to SCREENING 3]*
	[29] Norwich	*[Go to SCREENING 3]*
	[30] Port Dover	*[Go to SCREENING 3]*
	[31] Simcoe	*[Go to SCREENING 3]*
	[32] Tillsonburg	*[Go to SCREENING 3]*
	[33] Waterford	*[Go to SCREENING 3]*
	[130] Bealton	*[Go to PCODE]*
	[131] Boston	*[Go to PCODE]*
	[132] Canboro	*[Go to PCODE]*
	[133] Canfield	*[Go to PCODE]*
	[134] Fisherville	*[Go to PCODE]*
	[135] Langton	*[Go to PCODE]*
	[136] Long Point	*[Go to PCODE]*

	[137] Nanticoke [Go to PCODE]<br>[138] Normandale [Go to PCODE]<br>[139] Port Rowan [Go to PCODE]<br>[140] Selkirk [Go to PCODE]<br>[141] St. Williams [Go to PCODE]<br>[142] Teeterville [Go to PCODE]<br>[143] Townsend [Go to PCODE]<br>[144] Turkey Point [Go to PCODE]<br>[145] Villa Nova [Go to PCODE]<br>[146] Vittoria [Go to PCODE]<br>[147] Walsingham [Go to PCODE]<br>[148] York [Go to PCODE]<br><br>[ALWAYS DISPLAY]<br>[66] or another town or village? (please specify: _________________________)<br>*Proceed with Survey, periodically check responses post-survey]*<br>[77] Home is rural (outside a town or village, e.g., rural, agricultural or wilderness area)<br>*[Proceed with Survey]*<br>[-- DO NOT READ THE FOLLOWING ALOUD --]<br>[88] Don't know *[TERMINATE SURVEY]*<br>[99] Decline to Respond *[TERMINATE SURVEY]*<br><br>SCREENING3.<br>You appear to live in a community that is excluded from the survey area designated for this research. We are interested in talking to people who live in very small towns, villages or rural locations outside of towns.<br>Do you live **within** or **outside** the town limits of [TOWN SELECTED IN SCREENING2]?<br>[1] Within town limits *[TERMINATE SURVEY]*<br>[2] Outside town limits *[Probe and clarify. Go back to previous questions if necessary.]*<br>[88] Don't know [Probe]<br>[99] Decline to Respond [TERMINATE SURVEY]<br><br>**TERMINATE SURVEY FOR URBAN RURAL SCREENING.** In order to participate in the survey, you would need to live within the specific rural areas and towns we are interested in. We cannot proceed any further in the survey. Thank you for your time!<br><br>PCode<br>What is your postal code?<br>| - - - - - - | [allow 6]<br>[88] Don't know/Refused<br><br>**STATS BLOCK END**
ALL	**RANDOM2 determined the FORM A/B split**<br>**IF RANDOM2 < 50, administered FORM A**<br>**IF RANDOM2 >= 50, administered FORM B**
ALL	**INFANT DIARY BLOCK START**<br><br>An important part of this study is to learn what kinds of pollutants adults and children come in contact with in their daily activities. To do this we need to find out how and where people spend

	their time.<br><br>I would like to ask you about the things [fill name] did yesterday – from midnight [fill pday] night to midnight last night.<br><br>TYPE [1] to begin diary<br><br>THE FOLLOWING INFORMATION IS ASKED FOR EACH ACTIVITY:<br>1. START TIME OF ACTIVITY<br>2. DESCRIPTION OF ACTIVITY<br>3. WHERE ACTIVITY WAS DONE<br>4. END TIME OF ACTIVITY<br><br>When location codes 301-310, 313, and 315 are entered, a supplemental question will be asked:<br><br>QT<br>Was this activity conducted on or near a roadway with moderate to heavy traffic?<br><br>(Surveyor Note: A roadway with "moderate to heavy traffic" is one that has a substantial amount of traffic for several hours of the day, such as a main thoroughfare, a busy boulevard, or a highway. It does not include quiet residential streets or roads with occasional traffic.)<br><br>[1] NO<br>[2] YES *[QTa]*<br>[88] DK<br><br>QTA<br>About how much of this activity was conducted on or near a roadway with moderate to heavy traffic?<br>[0] LESS THAN 1 MINUTE<br>[1-120] RECORD THE NUMBER OF MINUTES<br>[180] 3 HOURS<br>[240] 4 HOURS<br>[300] 5 HOURS<br>[360] 6 HOURS<br>[420] 7 HOURS<br>[480] 8 HOURS<br>[540] 9 HOURS<br>[600] 10 HOURS<br>[660] 11 HOURS<br>[720] 12 HOURS<br>[800] MORE THAN 12 HOURS<br>[88] DK<br><br>*If possible, a built in check to compare the time spent in moderate to heavy traffic response to total time given for that activity would be desirable to ensure that the time in traffic does not exceed the time of the activity.*<br><br>**INFANT DIARY BLOCK END**
ALL	SURVEY CONSIDERED TO BE COMPLETE AFTER COMPLETING THE DIARY AS EARLY TERMINATION

ALL	**TRAFFIC BLOCK START**<br><br>TRAFFIC1<br>Thinking now about [fill NNAM] [fill ap] time spent outdoors yesterday, were there any times when [fill name] [fill were] in a car, van, truck or bus in moderate to heavy traffic?<br>[1] NO *[Traffic2]*<br>[2] YES<br>[88] DK *[Traffic2]*<br><br>TRAFFIC1A<br>For how long altogether?<br>[1] LESS THAN 1 MINUTE<br>[2] RECORD THE NUMBER OF MINUTES<br>[180] 3 HOURS<br>[240] 4 HOURS<br>[300] 5 HOURS<br>[360] 6 HOURS<br>[420] 7 HOURS<br>[480] 8 HOURS<br>[540] 9 HOURS<br>[600] 10 HOURS<br>[660] 11 HOURS<br>[720] 12 HOURS<br>[800] MORE THAN 12 HOURS<br>[88] DK<br><br>TRAFFIC2<br>Were there any times yesterday when [fill name] [fill were] near a road with moderate to heavy traffic?<br>[1] NO *[NEXT BLOCK]*<br>[2] YES<br>[88] DK *[NEXT BLOCK]*<br><br>TRAFFIC2A<br>For how long altogether?<br>[1] LESS THAN 1 MINUTE<br>[2] RECORD THE NUMBER OF MINUTES<br>[180] 3 HOURS<br>[240] 4 HOURS<br>[300] 5 HOURS<br>[360] 6 HOURS<br>[420] 7 HOURS<br>[480] 8 HOURS<br>[540] 9 HOURS<br>[600] 10 HOURS<br>[660] 11 HOURS<br>[720] 12 HOURS<br>[800] MORE THAN 12 HOURS<br>[88] DK<br><br>**TRAFFIC BLOCK END**
ALL	**PARKING BLOCK START**

	PARK1<br>Were there any times yesterday when [fill name] [fill were] in an enclosed parking garage or an indoor parking lot?<br>[1] NO *[Park2]*<br>[2] YES<br>[88] DK *[Park2]*<br><br>PARK1A<br>For how long altogether?<br>[1] LESS THAN 1 MINUTE<br>[2] RECORD THE NUMBER OF MINUTES<br>[180] 3 HOURS<br>[240] 4 HOURS<br>[300] 5 HOURS<br>[360] 6 HOURS<br>[420] 7 HOURS<br>[480] 8 HOURS<br>[540] 9 HOURS<br>[600] 10 HOURS<br>[660] 11 HOURS<br>[720] 12 HOURS<br>[800] MORE THAN 12 HOURS<br>[88] DK<br><br>PARK2<br>Were there any times yesterday when [fill name] [fill were] transported outside to a car in an open or outside parking area?<br>[1] NO *[NEXT BLOCK]*<br>[2] YES<br>[88] DK *[NEXT BLOCK]*<br><br>PARK2A<br>For how long altogether?<br>[1] LESS THAN 1 MINUTE<br>[2] RECORD THE NUMBER OF MINUTES<br>[180] 3 HOURS<br>[240] 4 HOURS<br>[300] 5 HOURS<br>[360] 6 HOURS<br>[420] 7 HOURS<br>[480] 8 HOURS<br>[540] 9 HOURS<br>[600] 10 HOURS<br>[660] 11 HOURS<br>[720] 12 HOURS<br>[800] MORE THAN 12 HOURS<br>[88] DK<br><br>**PARKING BLOCK END**
ALL	**GAS STATION BLOCK START**

	GSTAT1<br>Did [fill name] spend ANY time yesterday at a gas station or auto repair shop?<br>[1] NO *[NEXT BLOCK]*<br>[2] YES<br>[88] DK *[NEXT BLOCK]*<br><br>GSTAT2<br>About how long altogether yesterday did [fill name] spend in those places?<br>[1] LESS THAN 1 MINUTE<br>[2] RECORD THE NUMBER OF MINUTES<br>[180] 3 HOURS<br>[240] 4 HOURS<br>[300] 5 HOURS<br>[360] 6 HOURS<br>[420] 7 HOURS<br>[480] 8 HOURS<br>[540] 9 HOURS<br>[600] 10 HOURS<br>[660] 11 HOURS<br>[720] 12 HOURS<br>[800] MORE THAN 12 HOURS<br>[88] DK<br><br>GSTAT4<br>[fill were] [fill name] in a car when another person was pumping gasoline?<br>[1] NO<br>[2] YES<br>[88] DK<br>*[NEXT BLOCK]*<br><br>**GAS STATION BLOCK END**
ALL	**ENGINE BLOCK START**<br><br>ENGINE1<br>Yesterday, did any of [fill NNAM] [fill ap] activities at home, at day care or elsewhere involve working with or being near) Any gasoline or diesel-powered equipment, besides automobiles?<br>[1] NO<br>[2] YES *[Engine2]*<br>[88] DK<br>*[NEXT BLOCK]*<br><br>ENGINE2<br>About how long?<br>[1] LESS THAN 1 MINUTE<br>[2] RECORD THE NUMBER OF MINUTES<br>[180] 3 HOURS<br>[240] 4 HOURS<br>[300] 5 HOURS<br>[360] 6 HOURS<br>[420] 7 HOURS<br>[480] 8 HOURS<br>[540] 9 HOURS

	[600] 10 HOURS<br>[660] 11 HOURS<br>[720] 12 HOURS<br>[800] MORE THAN 12 HOURS<br>[88] DK<br><br>**ENGINE BLOCK END**
FORMA	**WATER BLOCK START**<br><br>WATER1<br>Do you get water for general household use from:<br>[1] THE PUBLIC WATER SYSTEM<br>[2] A PRIVATE WELL<br>[77] OR FROM SOME OTHER SOURCE – WHAT IS THAT? [specify]<br>[88] DK<br><br>WATER2<br>We are also interested in contact [fill name] had with tap water, that is, water from a faucet. First, how many times would you say [fill name] washed [fill your] hands yesterday?<br>[99] NONE<br>[1] 1-2<br>[2] 3-5<br>[3] 6-9<br>[4] 10-19<br>[5] 20-29<br>[6] 30+ TIMES<br>[88] DK<br><br>WATER4<br>Was [fill NAME] given a bath yesterday<br>[1] NO *[NEXT BLOCK]*<br>[2] YES *[Water4a]*<br>[88] DK *[NEXT BLOCK]*<br><br>WATER4A<br>How many baths was [fill NAME] given?<br>[1] RECORD ACTUAL NUMBER OF BATHS<br>[88] DK<br><br>WATER4B<br>How long did [fill name] spend being given the bath(s)? (IN TOTAL)<br>[1] RECORD ACTUAL MINUTES<br>[61] OVER AN HOUR<br>[88] DK<br><br>WATER4C<br>Was the bathroom door closed?<br>[1] NO<br>[2] YES<br>[88] DK<br><br>WATER4D

	Was there a window open or an exhaust fan on (in the bathroom)?<br>[1] NO<br>[2] YES<br>[88] DK<br><br>**WATER BLOCK END**
FORMA	**SOLVENT BLOCK START**<br><br>SOLVENT1<br>Yesterday, did any of [fill NNAM] [fill ap] activities at home, at day care or elsewhere involve working with or being near) Solvents, fumes or strong smelling chemicals?<br>[1] NO<br>[2] YES *[Solvent2]*<br>[88] DK<br>*[NEXT BLOCK]*<br><br>SOLVENT2<br>About how long?<br>[1] LESS THAN 1 MINUTE<br>[2] RECORD THE NUMBER OF MINUTES<br>[180] 3 HOURS<br>[240] 4 HOURS<br>[300] 5 HOURS<br>[360] 6 HOURS<br>[420] 7 HOURS<br>[480] 8 HOURS<br>[540] 9 HOURS<br>[600] 10 HOURS<br>[660] 11 HOURS<br>[720] 12 HOURS<br>[800] MORE THAN 12 HOURS<br>[88] DK<br><br>**SOLVENT BLOCK END**
ALL	**INFANT SMOKING BLOCK START**<br><br>PARSMOKE1<br>These next questions are about smoking. Did you or any members of your family or visitors smoke cigarettes in your home yesterday?<br><br>Surveyor: At home means both indoor and outdoor.<br><br>[1] NO *[ParSmoke3]*<br>[2] YES<br>[88] DK *[ParSmoke3]*<br><br>PARSMOKE2<br>About how many cigarettes altogether did you and they smoke at your home yesterday?<br><br>Surveyor: At home means both indoor and outdoor.<br><br>[1] 1-2

	[2] 3-5<br>[3] 6-9<br>[4] 10-14<br>[5] 15-24 (ONE PACK)<br>[6] 25-35<br>[7] 36+ (TWO PACKS OR MORE)<br>[88] DK<br><br>PARSMOKE3<br>Is smoking allowed in your home?<br>[1] NO<br>[2] YES<br>[88] DK<br><br>**INFANT SMOKING BLOCK END**
ALL	**HOUSE BLOCK 1 START**<br><br>BLOCK1<br>Is your home located on or within 1 block of a roadway with moderate to heavy traffic?<br>[1] NO<br>[2] YES<br>[88] DK<br><br>BLOCK1a<br>If [fill NAME] is cared for outside the home, is that location on or within 1 block of a roadway with moderate to heavy traffic<br>[2] INFANT IS NOT CARED FOR OUTSIDE THE HOME<br>[1] NO<br>[3] YES<br>[88] DK
FORMB	BLOCK2<br>Do you get water for general household use from:<br>[1] THE PUBLIC WATER SYSTEM<br>[2] A PRIVATE WELL<br>[77] OR FROM SOME OTHER SOURCE – WHAT IS THAT? [specify]<br>[88] DK
ALL	BLOCK3<br>Do you have an outdoor wood boiler?<br><br>Surveyor: An outdoor wood boiler also known as an outdoor wood furnace is a heating appliance that sits outside the home while providing heat to the home (see What IS Document for more information).<br><br>[1] NO<br>[2] YES<br>[88] DK<br><br>**HOUSE BLOCK 1 END**
ALL	**GARAGE BLOCK START**

	GARAGE1<br>Do you live in a house with an attached garage?<br><br>Surveyor: An apartment with underground parking garage does not qualify. A house, townhouse, or duplex qualifies.<br>[1] NO<br>[2] YES *[Garage2]*<br>[88] DK<br>*[NEXT BLOCK]*<br><br>GARAGE2<br>Is there a door connecting the garage to the home?<br>[1] NO *[Garage3]*<br>[2] YES *[Garage3]*<br>[88] DK *[Garage3]*<br><br>GARAGE3<br>Yesterday, was any automobile or other motor vehicle started in the garage?<br>[1] NO<br>[2] YES *[Garage4]*<br>[88] DK<br>*[NEXT BLOCK]*<br><br>GARAGE4<br>How many times? Would you say:<br><br>(read list)<br>[1] 1-2<br>[2] 3-5<br>[3] 6-9<br>[4] OR 10 OR MORE<br>[88] DK<br>*[NEXT BLOCK]*<br><br>GARAGE5<br>About how many of these times were the garage doors closed? Would you say:<br><br>SURVEYOR: This means the large garage doors to outside, not a door into the house. (read list)<br>[1] None<br>[2] 1-2<br>[3] 3-5<br>[4] 6-9<br>[5] OR 10 OR MORE<br>[88] DK<br>*[NEXT BLOCK]*<br><br>GOWI<br>I recorded that the car was started with the garage door closed more than the total number of times the car was started. Let me go back over these questions one more time.<br>TYPE [g] TO RETURN TO QUESTION Garage4 *[Garage4]*<br><br><br>**GARAGE BLOCK END**

FORMA	**STORAGE BLOCK START**<br><br>STORE1<br>Is any gasoline or kerosene being stored in any room or basement of your home or in an attached garage or carport?<br>[1] NO<br>[2] YES<br>[88] DK<br><br>STORE2<br>Are any devices with gasoline engines such as lawn mowers being stored in any room or basement of your home or in an attached garage or carport?<br>[1] NO<br>[2] YES<br>[88] DK<br><br>STORE3<br>Are any paints or varnishes being stored in any room or basement of your home or in an attached garage or carport?<br>[1] NO<br>[2] YES<br>[88] DK<br><br>STORE4<br>Are any mothballs, moth crystals or moth cakes used in your home?<br><br>Surveyor: Mothballs or other moth repellent are products containing chemical pesticide and deodorant used when storing clothing and other articles susceptible to damage from mold or moth larvae.<br><br>A moth is an insect related to butterfly and is commonly regarded as pests because their larvae eat fabric such as clothes and blankets made from natural fibers such as wool or silk.<br><br>[1] NO<br>[2] YES<br>[88] DK<br><br>**FORM A -STORAGE BLOCK END**
ALL	**AIR CONDITION BLOCK START**<br>**ASKED IN SUMMER ONLY**<br><br>AC1<br>Is your home air-conditioned?<br>[1] NO<br>[2] YES *[AC2]*<br>[88] DK<br>*[NEXT BLOCK]*<br><br>AC2<br>Do you use a central unit or a window or wall unit?<br>[1] CENTRAL<br>[2] WINDOW/WALL

	[88] DK<br><br>AC3<br>Was the air conditioning on at any time yesterday?<br>[1] NO<br>[2] YES *[AC4]*<br>[88] DK<br><br>AC4<br>Did [fill name] spend anytime at home yesterday while the air conditioning was on?<br>[1] NO<br>[2] YES<br>[88] DK<br><br>**AIR CONDITIONING BLOCK END**
ALL	**HEATING BLOCK START**<br>**ASKED IN WINTER ONLY**<br><br>HEAT1<br>Was any heat turned on at any time in your home yesterday?<br>[1] NO *[NEXT BLOCK]*<br>[2] YES *[Heat2]*<br>[88] DK *[NEXT BLOCK]*<br><br>HEAT2<br>Was a central furnace turned on?<br>[1] NO *[Heat5]*<br>[2] YES<br>[88] DK *[Heat5]*<br><br>HEAT3<br>What kind of fuel was used in a central furnace? (FIRST MENTION)<br>[1] GAS<br>[2] ELECTRICITY<br>[3] OIL<br>[4] COAL<br>[5] KEROSENE<br>[6] WOOD<br>[7] SOLAR<br>[77] OTHER (SPECIFY) [specify]<br>[88] DK *[Heat5]*<br><br>HEAT4<br>Any others? (SECOND MENTION)<br>[99] NO OTHERS/NONE<br>[1] GAS<br>[2] ELECTRICITY<br>[3] OIL<br>[4] COAL<br>[5] KEROSENE<br>[6] WOOD<br>[7] SOLAR

	[77] OTHER (SPECIFY) [specify]<br>[88] DK<br><br>HEAT5<br>Was any room [fill also] heated with a wood stove?<br>[1] NO<br>[2] YES<br>[88] DK<br>*[Heat6]*<br><br>HEAT6<br>Was any room [fill also] heated with a) Kerosene space heater?<br>[1] NO<br>[2] YES<br>[88] DK<br>*[Heat7]*<br><br>HEAT7<br>Was any room [fill also] heated with a) Electric space heater or electric baseboard heaters?<br>[1] NO<br>[2] YES<br>[88] DK<br>*[Heat8]*<br><br>HEAT8<br>Was any room [fill also] heated with a) Fireplace?<br>[1] NO<br>[2] YES<br>[88] DK<br>*[Heat9]*<br><br>HEAT9<br>Was any room [fill also] heated with a) Any other heat source?<br>[1] NO<br>[2] YES - What was that? [specify]<br>[88] DK<br><br>**HEATING BLOCK END**
FORMB	**HOUSE BLOCK 2 START**<br><br>Now, I have a few more questions about your home.<br><br>HOUSE 1<br>In what year did [fill name-child] move into your home?<br>[XXXX] RECORD ACTUAL YEAR<br>[88] DK/DON'T REMEMBER *[HOUSE2]*
ALL	[If location of survey is Annapolis Valley or Haldimand-Norfolk, GO TO Farm. If not, GO TO House2.]<br>FARM<br>Do you live on a farm?

	[1] NO<br>[2] YES<br>[88] DK<br><br>HOUSE2<br>Now, I have a few more questions about your home. Do you live in an:<br>[1] APARTMENT<br>[2] DETACHED SINGLE FAMILY HOUSE<br>[3] A TOWNHOUSE<br>[4] OR SOMETHING ELSE (SPECIFY) [specify]<br>[88] DK<br><br>HOUSE3<br>Was this building built:<br>[1] since 2001<br>[2] between 1991 and 2000<br>[3] between 1981 and 1990<br>[4] between 1971 and 1980<br>[5] between 1951 and 1970<br>[6] or earlier than 1951<br>[88] DK<br><br>HOUSE4<br>How many stories or floors are in your building or home?<br>(COUNT ONLY FLOORS WITH FINISHED ROOMS FOR LIVING PURPOSES OR FULL BASEMENTS)<br><br>A full or finished basement function as a habitable living space in the house. Typical examples include furnished recreation room, living room or a secondary suite.<br><br>An unfinished or partially finished basement serves primarily as a storage space. A basement with only a furnace, water tank, freezer, washer and dryer sets, or other storage would be considered an unfinished basement or partially finished basement.<br><br>[1] ONE<br>[2] TWO<br>[3] THREE<br>[4] 4 to 6<br>[5] 7 to 12<br>[6] 13 OR MORE STORIES<br>[88] DK
FORMB	HOUSE5<br>How many rooms (not counting bathrooms or half-rooms) do you have in your home?<br>[1] RECORD NUMBER<br>[31] MORE THAN 30<br>[88] DK *[House7]*<br><br>HOUSE6<br>Of these rooms, how many are carpeted or have rugs covering most of their surface?<br>** SHOULD BE LESS THAN OR EQUAL TO TOTAL ROOMS: [fill Q47/House5]<br>[1] NONE<br>[2] RECORD NUMBER

	[31] MORE THAN 30<br>[88] DK *[House7]*<br><br>[cowi] [if House5 eq [88] Then goto House7] [if House5 ge House6 then goto House7]<br><br>[COWI]<br>I recorded that you have more carpeted rooms than total rooms. Let me go back over these questions one more time.<br><br>** TYPE [g] TO RETURN TO HOUSE5<br><br>HOUSE7<br>Is there a basement in your building or house?<br>[1] NO<br>[2] YES<br>[88] DK<br><br>**HOUSE BLOCK 2 END**
FORMB	**RENOVATIONS BLOCK START**<br><br>RENO1<br>In the last six months, have you or anyone else renovated your home in any way? This would include indoor painting, refinishing floors, adding rooms to the house or laying new carpet.<br>[1] NO<br>[2] YES [Reno2]<br>[88] DK<br>*[NEXT BLOCK]*<br><br>RENO2<br>Was any indoor painting done?<br>[1] NO<br>[2] YES<br>[88] DK<br><br>RENO3<br>How about refinishing floors?<br>[1] NO<br>[2] YES<br>[88] DK<br><br>RENO4<br>What about additions to the house?<br>[1] NO<br>[2] YES<br>[88] DK<br><br>RENO5<br>What about laying carpet?<br>[1] NO *[NEXT BLOCK]*<br>[2] YES *[Reno6]*<br>[88] DK *[NEXT BLOCK]*

	RENO6<br>Was glue used or was it tacked down?<br>[1] GLUE<br>[2] TACKED<br>[77] OTHER<br>[88] DK<br><br>**RENOVATIONS BLOCK END**
FORMB	**FRESH AIR BLOCK START**<br><br>FRESH1<br>Does your home have a Heat Recovery Ventilator (HRV) unit?<br>[1] NO<br>[2] YES<br>[88] DK<br><br>FRESH2<br>Are exhaust fans used in the kitchen?<br>[1] NO<br>[2] YES<br>[88] DK<br><br>FRESH3<br>Are exhaust fans used in the bathroom?<br>[1] NO<br>[2] YES<br>[88] DK<br><br>FRESH4<br>Are exhaust fans used in the laundry?<br>[1] NO<br>[2] YES<br>[88] DK<br><br>FRESH5<br>Are open doors and windows used to provide fresh air to your home?<br>[1] NO<br>[2] YES<br>[88] DK<br><br>**FRESH AIR BLOCK END**
FORMB	**WINDOW AND DOOR BLOCK START**<br><br>WINDOW1<br>While [fill name] [fill were] at home yesterday, were any windows left open at all?<br>[1] NO<br>[2] YES *[Window2]*<br>[88] DK<br>*[Door1]*<br><br>WINDOW2<br>How many windows?

	[1] 1-2<br>[2] 3-5<br>[3] 6-9<br>[4] 10+<br>[88] DK *[Door1]*<br><br>WINDOW3<br>For about how long altogether were any windows left open?<br>[1] LESS THAN 1 MINUTE<br>[2] RECORD ACTUAL NUMBER OF MINUTES (LESS THAN 2 HOURS)<br>[180] FROM 2 TO 4 HOURS<br>[360] MORE THAN 4 UP TO 8 HOURS<br>[600] MORE THAN 8 UP TO 12 HOURS<br>[840] MORE THAN 12 UP TO 16 HOURS<br>[960] OVER 16 HOURS<br>[88] DK<br><br>DOOR1<br>Does your home have any doors which open directly to the outside of your building or house?<br>[1] NO *[NEXT BLOCK]*<br>[2] YES<br>[88] DK ***[NEXT BLOCK]***<br><br>DOOR2<br>While [fill name] [fill were] at home yesterday, were any of these outside doors left open at all?<br>[1] NO<br>[2] YES *[Door3]*<br>[88] DK<br>*[NEXT BLOCK]*<br><br>DOOR3<br>For about how long altogether was any outside door left open?<br>[1] LESS THAN 1 MINUTE<br>[2] RECORD ACTUAL NUMBER OF MINUTES (LESS THAN 2 HOURS)<br>[180] 2 HOURS TO 4 HOURS<br>[360] MORE THAN 4 UP TO 8 HOURS<br>[480] MORE THAN 8 UP TO 12 HOURS<br>[720] OVER 12 HOURS<br>[88] DK<br><br>DOOR4<br>While [fill name] [fill were] at home yesterday, about how many times would you say that an outside door was opened? Would you say:<br>[1] ONCE OR TWICE<br>[2] 3-5 TIMES<br>[3] 6-9 TIMES<br>[4] 10-19 TIMES<br>[5] OR 20 TIMES OR MORE<br>[88] DK<br><br>**WINDOW AND DOOR BLOCK END**
FORMB	**GAS OVEN BLOCK START**

	OVEN1<br>Do you have a gas range or gas oven in your home?<br>[1] NO<br>[2] YES *[Oven2]*<br>[88] DK<br>*[NEXT BLOCK]*<br><br>OVEN2<br>Does your gas range or gas oven have a continuously burning pilot light?<br>[1] NO<br>[2] YES<br>[88] DK<br><br>OVEN3<br>Did [fill name] spend any time yesterday at home when the gas range or gas oven was used?<br>[1] NO<br>[2] YES *[Oven4]*<br>[88] DK<br>*[NEXT BLOCK]*<br><br>OVEN4<br>For about how long [fill were] [fill name] home when the range or oven was on?<br>[1] LESS THAN 1 MINUTE<br>[2] RECORD THE NUMBER OF MINUTES<br>[180] 3 HOURS [480] 8 HOURS<br>[240] 4 HOURS [540] 9 HOURS<br>[300] 5 HOURS [600] 10 HOURS<br>[360] 6 HOURS [660] 11 HOURS<br>[420] 7 HOURS [720] 12 HOURS<br>[800] MORE THAN 12 HOURS<br>[88] DK<br><br>**GAS OVEN BLOCK END**
FORMA	**PESTICIDE BLOCK START**<br><br>PEST1<br>In the last six months, were any pesticides used to get rid of insects, rodents or other pests at your home?<br><br>Surveyor: Agricultural use of pesticides qualifies as yes.<br>[1] NO<br>[2] YES *[Pest2]*<br>[88] DK<br>*[NEXT BLOCK]*<br><br>PEST2<br>Was that done: (read list)<br><br>[1] INSIDE<br>[2] OUTSIDE<br>[3] OR BOTH?

	[88] DK<br><br>PEST3<br>How many of these times were pesticides applied by a PROFESSIONAL?<br>[1] NONE<br>[2] 1-2<br>[3] 3-5<br>[4] 6-9<br>[5] 10+ TIMES<br>[88] DK<br><br>PEST4<br>How many of these times did you PERSONALLY apply pesticides?<br>[1] NONE<br>[2] 1-2<br>[3] 3-5<br>[4] 6-9<br>[5] 10+ TIMES<br>[88] DK<br><br>**PESTICIDE BLOCK END**
ALL	**CHILD PLAY BLOCK START**<br><br>*If response to Qkid is 0, 6-17 or 99, go to NEXT BLOCK. If response to Qkid is 1-5, go to Play1.*<br><br>DIRT<br>I have a few more questions about [fill knam]. *Infant's name given at start of survey.*<br><br>PLAY1 Q39<br>Yesterday, did [fill dirt] spend any time crawling, lying, or playing on the floor in your home?<br>[1] NO<br>[2] YES *[Play1a]*<br>[88] DK<br>*[Play2]*<br><br>PLAY1A<br>Thinking of that floor area where [fill dirt] spent most of his or her time yesterday, is that area carpeted?<br>[1] NO, NOT CARPETED<br>[2] YES CARPETED<br>[88] DK<br><br>PLAY2<br>Did [fill dirt] spend any time yesterday playing outdoors?<br>[1] NO<br>[2] YES *[Play3]*<br>[88] DK<br>[99] REFUSED<br>*[NEXT BLOCK]*<br><br>PLAY3<br>When [fill dirt] was outdoors yesterday, did he/she spend any time playing on dirt?

	[1] NO<br>[2] YES *[Play3a]*<br>[88] DK<br>*[NEXT BLOCK]*<br><br>PLAY3A<br>About how much time was on dirt?<br>[1] LESS THAN 1 MINUTE<br>[2] RECORD THE NUMBER OF MINUTES<br>[180] 3 HOURS<br>[240] 4 HOURS<br>[300] 5 HOURS<br>[360] 6 HOURS<br>[420] 7 HOURS<br>[480] 8 HOURS<br>[540] 9 HOURS<br>[600] 10 HOURS<br>[660] 11 HOURS<br>[720] 12 HOURS<br>[800] MORE THAN 12 HOURS<br>[88] DK<br><br>**CHILD PLAY BLOCK END**
ALL	**HEALTH BLOCK START**<br><br>HEALTH1<br>Next, I have a few questions about [fill NAME-child] health. Has a doctor told you that [fill name] [fill have] asthma?<br>[1] NO<br>[2] YES<br>[88] DK<br><br>HEALTH2<br>Has a doctor told you that [fill NAME-child] [fill have] heart disease?<br>[1] NO<br>[2] YES<br>[88] DK<br><br>HEALTH3<br>Has a doctor told you that [fill NAME-child] [fill have] Chronic bronchitis or emphysema?<br>[1] NO<br>[2] YES<br>[88] DK<br><br>HEALTH4<br>Does a long-term physical condition, mental condition, or health problem, reduce the amount or kind of activity [fill NAME] can do? A “long-term condition” refers to a condition that is expected to last or has already lasted 6 months or more.<br>[1] SOMETIMES<br>[2] OFTEN<br>[3] NEVER<br>[88] DK

	[99] REF<br><br>**HEALTH BLOCK END**
ALL	**INCOME BLOCK START**<br><br>INCQ1<br>I now have a question regarding your household income. Please be assured that, like all other information you have provided, these answers will be kept strictly confidential. What is your best estimate of the total income, before taxes and deductions, of all household members from all sources in the past 12 months?<br>[allow 7] [MIN: 0] [MAX: 1,000,000; warning after 250,000, go to QINCQ1TEST]<br>[1] | - - - - - - | [2] $0 *[VERF]*<br>[3] MORE THAN $500,000 *[VERF]*<br>[88] DK/REF *[INCQ2]*<br><br>QINCQ1TEST<br>You have said that your household income is [$]. Is this correct?<br><br>INCQ2<br>Can you estimate in which of the following groups your household income falls?<br><br>[1] $0 to $5,000 *[VERF]*<br>[2] $5,001 to $10,000 *[VERF]*<br>[3] $10,001 to $15,000 *[VERF]*<br>[4] $15,001 to $20,000 *[VERF]*<br>[5] $20,001 to $30,000 *[VERF]*<br>[6] $30,001 to $40,000 *[VERF]*<br>[7] $40,001 to $50,000 *[VERF]*<br>[8] $50,001 to $60,000 *[VERF]*<br>[9] $60,001 to $80,000 *[VERF]*<br>[10] $80,001 to $100,000 *[VERF]*<br>[11] $100,001 or more *[VERF]*<br>[88] DK *[VERF]*<br>[99] RF *[VERF]*<br><br>**INCOME BLOCK END**
ALL	**VERIFICATION BLOCK START**<br><br>VERF<br>And, finally, your number is [fill racd] [fill tel1]-[fill tel2:0]?<br><br>[1] NO - What number have I reached? (SPECIFY) [specify]<br>[2] YES<br>[99] REF<br><br>**VERIFICATION BLOCK END**
ALL	**DRAW BLOCK START**<br><br>DRAW1<br>By completing this survey, you are eligible for entry in a draw to win a cash prize valued at $300. Would you like to enter into the prize draw?

	[1] YES *[CONTACT1]*<br>[2] NO *[END]*<br><br>CONTACT1<br>Please provide your first name and the best telephone number(s) to reach you should you be randomly selected as the winner.<br><br>Any information you provide will be stored for the purpose of the draw only. Your name and phone number will not be associated with your survey responses. All contact information will be destroyed after the draw has taken place and winners notified.<br><br>[1] First Name ______________________________ *[END]*<br>[2] Home Phone_____________________________ *[END]*<br>[3] Cell Phone______________________________ *[END]*<br>[4] Other Phone_____________________________ *[END]*<br><br>**DRAW BLOCK END**

Appendix B – CHAPS 2 Activity and Location Codes

Table B.1 Non-Infant Activity Codes

General Category	*Code*	*Activity*
Work Related	1	Main Job
	2	Unemployment
	3	Travel During Work
	5	Second Job
	8	Breaks
	9	Travel To/From Work
Household Work	10	Food Preparation
	100	Cooking Food on Range or Stove
	11	Food Cleanup
	12	Cleaning House
	13	Outdoor Cleaning
	14	Clothes Care
	15	Car Repair/Maintenance
	16	Other Repairs (By Respondent)
	17	Plant Care
	18	Animal Care
	19	Other Household Work
Child Care	20	Baby Care
	21	Child Care
	22	Helping/Teaching
	23	Talking/Reading to Children
	24	Indoor Playing
	25	Outdoor Playing
	26	Medical Care-Child
	27	Other Child Care
	29	Travel for Child Care
Obtaining Goods	30	Shopping for Food
	31	Shopping for Clothes/HH Items
	28	Dry Cleaning
	32	Personal Care Services
	33	Medical Appointments
	34	Govt/Financial Services
	35	Car Repair Services
	36	Other Repair Services
	37	Other Services
	38	Errands
	39	Travel Related to Goods and Services
Personal Needs	40	Washing (e.g., Showering, Bathing)
	41	Medical Care
	42	Help and Care
	43	Eating
	44	Personal Hygiene
	45	Sleeping/Napping
	47	Dressing, etc.
	48	Other (Not ascertained) Activities
	49	Travel for Personal Care

General Category	*Code*	*Activity*
Educational	50	Attending F/T School
	51	Other Classes
	54	Doing Homework
	55	Using Library
	56	Other Education
	59	Travel for Education
	591	Recess/Break
Organizational	60	Professional/Union
	61	Special Interest
	62	Political/Civic
	63	Volunteer/Helping
	64	Religious Groups
	65	Religious Practices (By R)
	66	Fraternal Organization
	67	Child/Youth/Family Organization
	68	Other Organizations
	69	Travel for Organizational Activities
Social, Entertainment	70	Sports Events
	71	Entertainment
	72	Cinema/Movies
	73	Theatre
	74	Museums
	75	Visiting
	76	Parties
	77	Bars/Lounges
	78	Other Social
	79	Travel for Social
Recreation	80	Active Sports
	81	Outdoor
	82	Exercise
	83	Hobbies
	84	Domestic Crafts
	85	Art
	86	Music/Drama/Dance
	87	Board/Card Games
	88	Computer Use / Video Games
	89	Travel for Recreation Activities
Media, Communications, Passive Leisure	90	Radio
	91	TV / DVD
	92	Records/Tapes/CDs/Digital Music
	93	Reading Books
	94	Reading Magazines/etc.
	95	Reading Newspaper
	96	Conversations / Talking / Texting
	97	Letters, Writing, Paperwork
	98	Thinking/Relaxing
	99	Travel Related to Passive Leisure
Travel Related Activity	101	Travel to Home

Table B.2 Infant Activity Codes

General Category	*Code*	*Activity*
Personal Needs	431	Being Bottle-Fed
	432	Being Breast-Fed
	46	Crying/Fussing
	47	Dressing, Grooming, Diaper Change
	433	Drinking
	43	Eating
	44	Personal Hygiene
	45	Sleeping/Napping
	40	Washing (E.G., Bathing)
	48	Other (Not Ascertained) Activities
Child Care	271	Cuddling/Rocking/Soothering
	33	Medical Appointments
	20	Receiving Baby Care
	26	Receiving Medical Care
	27	Other Child Care
Playing	85	Art (E.G., Paints And Crayon)
	82	Exercise (E.G., Swimming, Bouncing, Crawling)
	24	Indoor Playing
	25	Outdoor Playing
	241	Playing With Dirt/Plant
	242	Playing With Other
	243	Playing With Pet/Animal
	244	Playing With Toy
	248	Other Playing
Learning	1001	Exploring
	1002	Imitating/Stimulating
	1003	Interacting With Object/Things
	1008	Other Learning
Educational, Organizational	67	Child/Youth/Family Organization
	52	Daycare Or Nursery
	65	Religious
	56	Other Education
	68	Other Organizational
Recreation, Leisure	982	Awake And Content
	932	Being Read To
	96	Conversations / Talking
	93	Looking At Book/Magazines
	90	Radio
	92	Records/Tapes/CDs/Digital Music
	981	Thinking/Relaxing/Being Sang To
	91	TV / DVD
	988	Other Recreation
	983	In a stroller or a carrier
Social, Entertainment	781	Dancing
	71	Entertainment
	782	Helping
	74	Museums
	78	Other Social

General Category	*Code*	*Activity*
	76	Parties
	70	Sports Events
	75	Visiting
Travel Related Activity	101	Travel To Home
	9	Travel To/From Work (With Parent/Guardian)
	29	Travel For Child Care
	39	Travel For Goods And Services
	49	Travel For Personal Need
	59	Travel For Education Or Organizational Activities
	249	Travel For Playing
	89	Travel For Recreation
	79	Travel For Social, Entertainment
	1009	Travel For Learning
	299	Transported Into And Out Of Vehicle (Include Strapping Infant Into Car Seat)

Table B.3 Location Codes

General Category	*Code*	*Location*
In Own Home	100	Home-Other
	101	Kitchen
	102	Living Room/Family Room/Den
	103	Dining Room
	104	Bathroom
	105	Bedroom
	106	Study/Office
	107	Garage
	108	Basement
	110	Utility Room/Laundry Room
	111	Pool, Spa (outdoors)
	112	Yard, Patio, Other Outside House
	113	Moving from Room to Room
	114	Moving In and Out of the House
	120	Other Verified
	199	Refused
In Other's House	200	Home-Other
	201	Kitchen
	202	Living Room/Family Room/Den
	203	Dining Room
	204	Bathroom
	205	Bedroom
	206	Study/Office
	207	Garage
	208	Basement
	210	Utility Room/Laundry Room
	211	Pool, Spa (outdoors)
	212	Yard, Patio, Other Outside House
	213	Moving from Room to Room
	214	Moving In and Out of the House
	220	Other Verified
	299	Refused
Traveling	300	Other Travel Location Specify
	301	Car
	302	Truck (Pick-up or van)
	303	Truck (Other than pick-up or van)
	304	Motorcycle/Moped/Motorized Scooter
	305	Bus
	306	Walking
	307	Bicycle/Skateboard/Roller-Skates
	308	In a Stroller/Carried by an Adult
	310	Train/Subway/Rapid Transit
	311	Airplane
	312	Boat
	313	Waiting for Bus, Train, Ride (at stop)
	314	Waiting for Travel, Indoors
	315	Running/Jogging
	320	Other Verified

General Category	*Code*	*Location*
	399	Refused
Other Indoor	400	Other Indoor-Specify
	401	Office Building/Bank/Post Office
	402	Industrial Plant/Factory/Warehouse
	403	Grocery Store/Convenience Store
	404	Shopping Mall/Non-Grocery Store
	405	Bar/Night Club/Bowling Alley
	406	Auto Repair Shop/Gas Station
	407	Indoor Gym/Sports or Health Club
	408	Public Building/Library/Museum/Theatre
	409	Laundromat
	410	Hospital/Health Care Facility/Doctor's Office
	411	Beauty Parlour/Barber Shop/Hair Dressers
	412	At Work: No Specific Main Location, Moving Among Locations
	413	School
	414	Restaurant
	415	Church
	416	Hotel/Motel
	417	Dry Cleaners
	418	Other Repair Shop
	419	Indoor Parking Garage
	420	Other Indoor Verified
	421	Indoor Pool
	499	Refused
Other Outdoor	500	Other Outdoor Specify
	501	Sidewalk/Street/Neighbourhood
	502	Parking Lot
	503	Service Station/Gas Station
	504	Construction Site
	505	School Grounds/Playground
	506	Sports Stadium
	507	Park/Golf Course
	508	River, Lake
	510	Restaurant/Picnic
	511	Farm
	512	Outdoor Pool
	520	Other Verified
	599	Refused

Appendix C – Location Groupings

Analysis Group	*Code*	*Location*
Indoors at home	100	Home-Other
	101	Kitchen
	102	Living Room/Family Room/Den
	103	Dining Room
	104	Bathroom
	105	Bedroom
	106	Study/Office
	107	Garage
	108	Basement
	110	Utility Room/Laundry Room
	113	Moving from Room to Room
	114	Moving In and Out of the House
	120	Other Verified
	199	Refused
Other indoor locations	200	Home-Other
	201	Kitchen
	202	Living Room/Family Room/Den
	203	Dining Room
	204	Bathroom
	205	Bedroom
	206	Study/Office
	207	Garage
	208	Basement
	210	Utility Room/Laundry Room
	213	Moving from Room to Room
	214	Moving In and Out of the House
	220	Other Verified
	299	Refused
	314	Waiting for Travel, Indoors
	400	Other Indoor-Specify
	401	Office Building/Bank/Post Office
	402	Industrial Plant/Factory/Warehouse
	403	Grocery Store/Convenience Store
	404	Shopping Mall/Non-Grocery Store
	405	Bar/Night Club/Bowling Alley
	406	Auto Repair Shop/Gas Station
	407	Indoor Gym/Sports or Health Club
	408	Public Building/Library/Museum/Theatre
	409	Laundromat
	410	Hospital/Health Care Facility/Doctor's Office
	411	Beauty Parlour/Barber Shop/Hair Dressers
	412	At Work: No Specific Main Location, Moving Among Locations
	413	School
	414	Restaurant
	415	Church
	416	Hotel/Motel
	417	Dry Cleaners
	418	Other Repair Shop

Analysis Group	*Code*	*Location*
	419	Indoor Parking Garage
	420	Other Indoor Verified
	421	Indoor Pool
	499	Refused
Outdoors	111	Pool, Spa (outdoors) at own home
	112	Yard, Patio, Other Outside House
	211	Pool, Spa (outdoors) at other's home
	212	Yard, Patio, Other Outside House
	306	Walking
	307	Bicycle/Skateboard/Roller-Skates
	308	In a Stroller/Carried by an Adult
	313	Waiting for Bus, Train, Ride (at stop)
	315	Running/Jogging
	500	Other Outdoor Specify
	501	Sidewalk/Street/Neighbourhood
	502	Parking Lot
	503	Service Station/Gas Station
	504	Construction Site
	505	School Grounds/Playground
	506	Sports Stadium
	507	Park/Golf Course
	508	River, Lake
	510	Restaurant/Picnic
	511	Farm
	512	Outdoor Pool
	520	Other Verified
	599	Refused
In vehicle	300	Other Travel Location Specify
	301	Car
	302	Truck (Pick-up or van)
	303	Truck (Other than pick-up or van)
	304	Motorcycle/Moped/Motorized Scooter
	305	Bus
	310	Train/Subway/Rapid Transit
	311	Airplane
	312	Boat
	320	Other Verified
	399	Refused
